# Incorporation of paramagnetic, fluorescent and PET/SPECT contrast agents into liposomes for multimodal imaging

**DOI:** 10.1016/j.biomaterials.2012.09.070

**Published:** 2013-01

**Authors:** Nick Mitchell, Tammy L. Kalber, Margaret S. Cooper, Kavitha Sunassee, Samantha L. Chalker, Karen P. Shaw, Katherine L. Ordidge, Adam Badar, Samuel M. Janes, Philip J. Blower, Mark F. Lythgoe, Helen C. Hailes, Alethea B. Tabor

**Affiliations:** a Department of Chemistry, University College London, Christopher Ingold Laboratories, 20 Gordon St, London WC1H 0AJ, UK; b Centre of Advanced Biomedical Imaging, Division of Medicine and Institute of Child Health, University College London, 72 Huntley Street, WC1E 6DD, UK; c Centre for Respiratory Research, University College London, Rayne Building, 5 University Street, WC1E 6JJ, UK; d King's College London, St. Thomas' Hospital, Division of Imaging Sciences and Biomedical Engineering, 4th Floor, Lambeth Wing, St Thomas' Hospital, London SE1 7EH, UK; e Royal Institution of Great Britain, Davy Faraday Research Laboratories, 21 Albemarle Street, London W1S 4BS, UK; f King's College London, Division of Chemistry, Hodgkin Building, Guy's Campus, London SE1 1UL, UK

**Keywords:** DOTA-lipid, Liposome, MRI (magnetic resonance imaging), PEG (poly(ethylene)glycol), SPECT (single-photon emission tomography), DCC, *N,N*-dicyclohexylcarbodiimide, DEG1SL, dioleylethyleneglycol-1-succidimidyl linker, DEG3SL, dioleylethyleneglycol-3-succidimidyl linker, DEG6SL, dioleylethyleneglycol-6-succidimidyl linker, DODEG4, DiOleylDimethyl Ethylene Glycol 4, DOPE, 1,2-dioleoyl-*sn*-glycero-3-phosphoethanolamine, DOTA, 1,4,7,10-tetraazacyclododecane-1,4,7,10-tetraacetic acid, DOTMA, *N*-[1-(2,3-dioleyloxy)propyl]-*N,N,N*-trimethylammonium chloride, DSPE-PEG2000, 1,2-distearoyl-*sn*-glycero-3-phosphoethanolamine-*N*-[carboxy(polyethyleneglycol)_2000_], DTPA, diethylenetriamine pentacetic acid, *n*-EG, *n*-ethylene glycol, EPR, enhanced permeability and retention effect, FL-DHPE, *N*-(fluorescein-5-thiocarbamoyl)-1,2-dihexa-decanoyl-sn-glycero-3-phosphoethanolamine, HBTU, *O*-(benzotriazol-1-yl)-*N,N,N′,N′*-tetramethyluronium hexafluorophosphate, ITLC, instant thin layer chromatography, MR, magnetic resonance, PEG, polyethylene glycol, PET, positron emission tomography, RES, reticuloendothelial system, SPECT, single-photon emission tomography

## Abstract

A series of metal-chelating lipid conjugates has been designed and synthesized. Each member of the series bears a 1,4,7,10-tetraazacyclododecane-1,4,7,10-tetraacetic acid (DOTA) macrocycle attached to the lipid head group, using short *n*-ethylene glycol (*n*-EG) spacers of varying length. Liposomes incorporating these lipids, chelated to Gd^3+^, ^64^Cu^2+^, or ^111^In^3+^, and also incorporating fluorescent lipids, have been prepared, and their application in optical, magnetic resonance (MR) and single-photon emission tomography (SPECT) imaging of cellular uptake and distribution investigated *in vitro* and *in vivo*. We have shown that these multimodal liposomes can be used as functional MR contrast agents as well as radionuclide tracers for SPECT, and that they can be optimized for each application. When shielded liposomes were formulated incorporating 50% of a lipid with a short *n*-EG spacer, to give nanoparticles with a shallow but even coverage of *n*-EG, they showed good cellular internalization in a range of tumour cells, compared to the limited cellular uptake of conventional shielded liposomes formulated with 7% 1,2-distearoyl-*sn*-glycero-3-phosphoethanolamine-*N*-[carboxy(polyethyleneglycol)_2000_] (DSPE-PEG2000). Moreover, by matching the depth of *n*-EG coverage to the length of the *n*-EG spacers of the DOTA lipids, we have shown that similar distributions and blood half lives to DSPE-PEG2000-stabilized liposomes can be achieved. The ability to tune the imaging properties and distribution of these liposomes allows for the future development of a flexible tri-modal imaging agent.

## 1 Introduction

The use of liposomes for the targeted delivery of therapeutic small molecules, DNA, or siRNA to tumours has generated a great deal of interest in the cancer research community [1]. Liposomes are versatile nanoparticles with low toxicity that can be formulated from a range of natural and biologically inspired synthetic lipids. They have been extensively used in the clinic as drug carriers to reduce the toxicity of potent drugs, such as doxorubicin, to non-cancerous cells and tissues. Hydrophilic drugs can be trapped in the central aqueous core of the liposomes, and lipophilic drugs can be solubilised within the lipid bilayer [1]: in either case, the drug cannot readily pass through the lipid bilayer and is trapped within the liposome until the nanoparticle reaches the tumour. For successful *in vivo* applications, it has been established that the outer bilayer of the liposome should ideally be coated with a neutral polyethylene glycol (PEG) polymer to minimize colloidal instability, reducing bioadhesion and limiting immunological responses [2]. Most importantly, the PEG coating reduces uptake of the liposome within the reticuloendothelial system (RES) and therefore slows the rate of removal of the liposomes from the blood [2]. This effectively increases the biological half-life of the liposome; in clinical studies conventional liposomes have been shown to have a half-life of 20 min in body fluids, whereas PEG-liposomes can have a half-life of up to 5 days [3], allowing for greater payloads of drug to be delivered to the tumour before clearance. Liposomal formulations of anticancer drugs have now been approved for clinical use: DOXIL^®^ (PEGylated liposomal doxorubicin) is used to treat AIDS-related Kaposi's sarcoma and multiple myeloma [4] and is in clinical trials for the treatment of breast cancer [1].

The PEG coating of liposomes is also highly advantageous in terms of passive targeting to tumours. Tumour vasculature is characterized by a chaotic network of thin-walled, leaky vessels [5] and therefore liposomes are able to cross into the interstitial spaces in viable tumour areas with limited wash out, a process referred to as the enhanced permeability and retention (EPR) effect [6]. Small PEG-liposomes, 100–200 nm in diameter, are the right size to permeate through the tumour vasculature, and have been shown to accumulate in tumour tissue. However, liposomal particles with high percentages of long chain PEG, such as PEG2000, at the ratios needed for *in vivo* applications, have shown limited uptake in cells *in vitro* [7]. This problem is particularly acute when liposomes are used to deliver DNA or siRNA, as the resulting PEG-coated lipoplexes are also frequently too stable to disassemble once internalized in the endosome [8]. Targeting moieties, such as ligands for receptor mediated uptake, have therefore been used to enhance the uptake of PEG-liposomes [7], although this approach is not always effective [9].

We have previously developed ternary lipid-peptide-DNA lipopolyplex vectors that have been formulated with cationic lipids bearing short *n*-ethylene glycol (*n*-EG) units (e.g. *n* = 4: DODEG4 (Fig. 2), or *n* = 6), based on the structure of (*N*-[1-(2,3-dioleyloxy)propyl])-*N,N,N*-trimethylammonium chloride (DOTMA) [10,11]. These lipopolyplexes also incorporate multifunctional peptides comprising both a cationic sequence for the complexation of plasmid DNA and an integrin-binding sequence to target the nanoparticles to cells of interest [11]. We have co-formulated the *n*-EG cationic lipids with 1,2-dioleoyl-*sn*-glycero-3-phosphoethanolamine (DOPE) in a 1:1 (w:w) ratio of cationic lipid:DOPE, to give a vector which we hypothesise is shielded by an even coverage of short *n*-ethylene glycol units. These shielded lipopolyplexes were demonstrated to be compact, stable under physiologically relevant conditions, and maintain tumour-specific targeting and transfection properties *in vitro* [11] and *in vivo* [12]. In contrast, in previously reported studies of PEG-liposomes [2], the maximum possible loading of shielding lipids such as DSPE-PEG2000 is about 10% of PEG-lipid, resulting in an incomplete surface coverage.

Multifunctional nanoparticles that combine the delivery of therapeutic agents with the capacity for *in vivo* imaging are attractive as they enable simultaneous monitoring and treatment of diseases. In recent years there has been considerable interest in the development of such theranostic nanoparticles [13], which allow the distribution, selectivity, efficacy and uptake of the therapeutic agent in target tissues to be determined. In previous research, liposomal delivery of therapeutic agents has been combined with imaging techniques such as magnetic resonance imaging (MRI) [13–20], positron or single-photon emission tomography (PET/SPECT) [21–23] and fluorescence [13–17]. These imaging methods are currently at the forefront of medical diagnostics and widely used in the preclinical and clinical settings for assessment of treatment efficacy. MRI is a non-invasive and widely used imaging modality that produces excellent soft tissue contrast and spatial resolution. However, MRI suffers from poor sensitivity, and therefore the target to be imaged either has to be expressed at a high level, or approaches to increase the contrast payload are needed [14,24]. In contrast, nuclear imaging with PET and SPECT methods provides exquisite sensitivity but has limited spatial and temporal resolution [25]. Therefore, there is a need to combine these imaging modalities in order to elucidate the biological behaviour of liposomes from the cellular level to the macroscopic scale. A drug delivery system that incorporates reporter groups for these imaging modalities would be highly desirable, and a multimodal approach that encompasses all three would be ideal, as it would allow the limitations of each imaging modality to be overcome [26].

One of the most straightforward approaches for the incorporation of both the MRI contrast agent Gd^3+^, and appropriate radionucleides for PET/SPECT nuclear imaging, into liposomes, is to use a chelating ligand, suitably modified for attachment to an organic scaffold or small molecule [27,28]. An ideal chelator for these purposes is the macrocycle 1,4,7,10-tetraazacyclododecane-1,4,7,10-tetraacetic acid (DOTA), which has been used extensively for the chelation of a range of metal ions that can provide MRI contrast or PET/SPECT imaging. Due to its cyclic nature, the dissociation of the metal ion from the complex is very slow, especially when compared to linear chelators such as diethylenetriamine pentacetic acid (DTPA), significantly lowering the toxicity of DOTA-chelated contrast agents [29]. Gd.DOTA (Dotarem^®^) is clinically approved for use in MRI and has been used previously for the attachment of Gd-chelates to the head group of various lipids [27]. The nature of the groups attached to the DOTA, flexibility of the linker between the liposome and the chelate, and the size of the resulting complex, can all be used to tune the sensitivity of the resulting contrast agent [27,28]. Similarly, liposomes labelled with a wide range of radionuclides have been reported for PET and SPECT nuclear imaging, and for targeted radiotherapy [30], although the majority of these radioactive liposome formulations rely on the encapsulation of radionuclide chelates rather than the inclusion of chelating lipids.

**Fig. 1:**
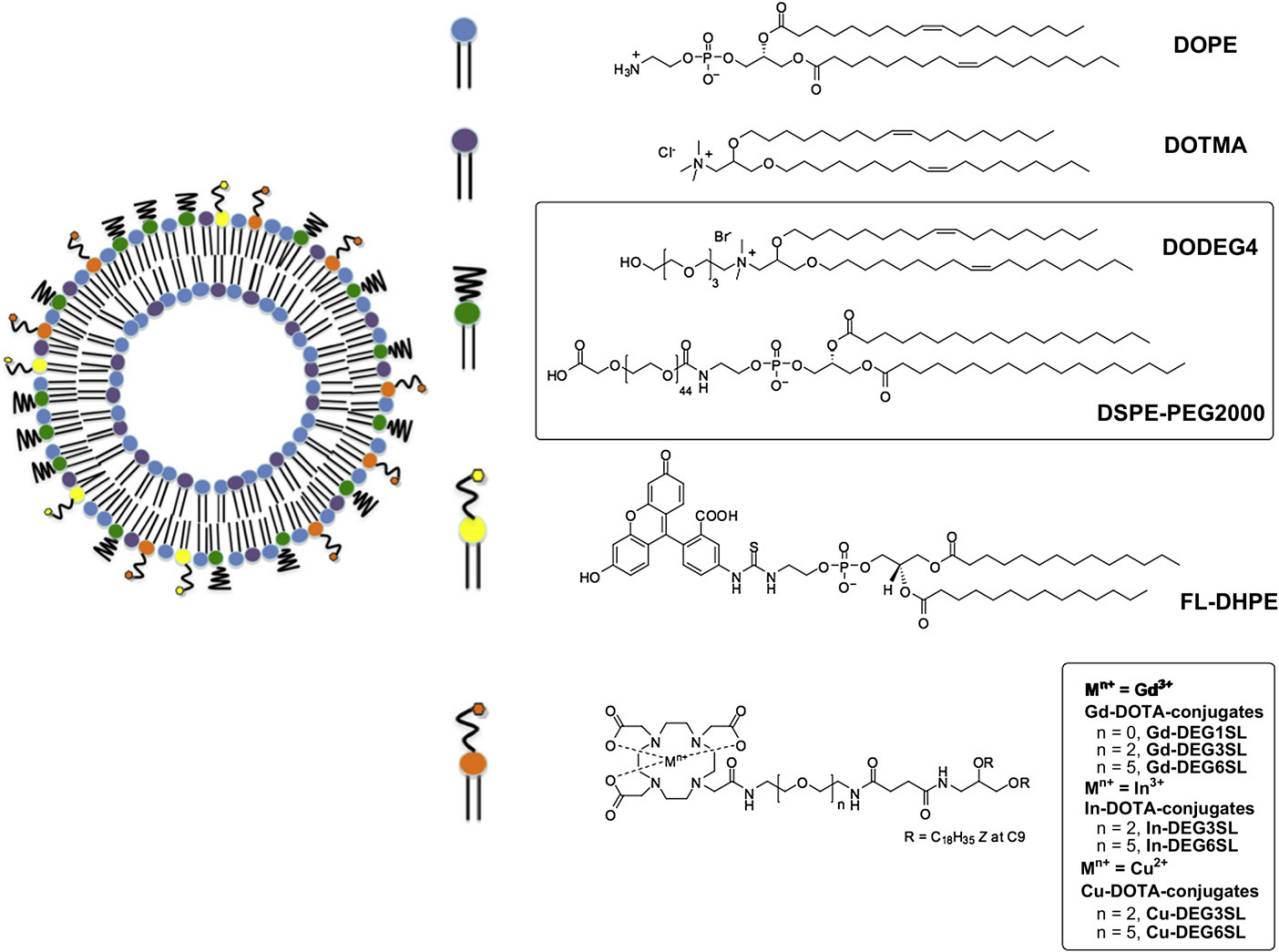
Neutral, cationic and PEG-lipids used in this study. General structure of DOTA-lipids chelated to paramagnetic or radioactive ions, and schematic showing the multifunctional liposomes formulated.

The aim of this study was to develop a multifunctional, multimodal shielded liposomal formulation, incorporating: lipids that have short *n*-EG units to shield the liposomes *in vivo*; DOTA-lipids with short *n*-EG units which can chelate paramagnetic ions and/or radionuclides; a fluorophore-labelled lipid to allow for optical imaging; and the helper lipid DOPE (Fig. 1). We have designed and synthesized three lipids with DOTA attached at the head group, and with different length *n*-EG spacer units between DOTA and the lipid acyl chains. Chelation of paramagnetic (Gd^3+^) ions to the conjugated DOTA, will allow these liposomes to be used for the development of imaging tracers for MRI. Similarly, chelation of radioactive ions to the DOTA, post-formulation, will lead to tracers for PET or SPECT. ^64^Cu was selected for these studies. This is increasingly seen as the radionuclide of choice for *in vivo* PET studies as it has a relatively long half-life (12.7 h); gives high-quality PET images, and can also be used in radionuclide therapy [31]. Similarly, ^111^In has a long half life (2.8 days) and is in clinical use for several applications [32]. Both radionuclides are effectively chelated by DOTA with good *in vivo* kinetic stability.

It was particularly important when developing these systems to compare the biodistribution characteristics of shielded liposomes formulated with varying amounts of lipids bearing short *n*-EG units, to that of liposomes formulated with PEG2000-bearing lipids to enable future targeting applications for drug delivery and diagnostic imaging.

## 2 Materials and methods

### 2.1 Materials

1,2-dioleoyl-*sn*-glycero-3-phosphoethanolamine (DOPE), 1,2-distearoyl-*sn*-glycero-3-phosphoethanolamine-*N*-[carboxy(polyethyleneglycol)_2000_], ammonium salt (DSPE-PEG2000) and *N*-(fluorescein-5-thiocarbamoyl)-1,2-dihexa-decanoyl-*sn*-glycero-3-phosphoethanolamine, triethylammonium salt (FL-DHPE) were purchased from Avanti Polar Lipids Inc. (Alabaster, AL). [^111^In]-InCl_3_ was purchased from Covidien Commercial Ltd, Hants UK. [^64^Cu]-CuCl_2_ was produced by proton bombardment of enriched Ni-64 metal and purified as previously described using Fraction 9 [33]. All other reagents were purchased from Sigma–Aldrich Co. Ltd. unless otherwise stated, and used without further purification. All reagents were of commercial quality and used as received and all solvents anhydrous. Thin Layer Chromatography (TLC) was performed on aluminium backed Sigma–Aldrich TLC plates with F_254_ fluorescent indicator. Visualisation was done by quenching of UV fluorescence or by staining the plates with potassium permanganate solution [KMnO_4_ (1.25 g), Na_2_CO_3_ (6.25 g), water (250 mL)]. ITLC-SA plates were from Varian/Agilent. Normal phase flash chromatography was carried out using silica gel (43–60 μm) supplied by Merck. LC/MS was performed on a Waters Acquity uPLC SQD using HPLC grade water and acetonitrile (both with 0.1% formic acid) as the solvents. MALDI MS was performed on a Waters MALDI MicroMX machine using α-cyano-4-hydroxycinnamic acid (CHCA) or sinapinic acid (SA) as the matrix (1 mg/mL in methanol). NMR (^1^H and ^13^C) was performed on either 500 or 600 MHz AMX Bruker Spectrometers (as stated). The chemical shifts (*δ*) were given in units of ppm relative to tetramethylsilane (TMS), where *δ* (TMS) = 0 ppm. Coupling constants (*J*) were measured in Hertz (Hz), multiplicities for ^1^H coupling are shown as s (singlet), d (doublet), t (triplet), m (multiplet), or a combination of the above. Deuterated chloroform (CDCl_3_), dimethylsulfoxide (d_6_-DMSO) and methanol (CD_3_OD) were used as solvents (as stated) for all NMR analysis. Dynamic light scattering and zeta potential measurements were performed using a Malvern Zetasizer Nano-ZS (Malvern, UK). MRI experiments were conducted on a Agilent 9.4 T scanner (Agilient Inc. Palo Alto, CA, USA) using a 39 mm coil (RAPID Biomed, Rimpar, Germany). SPECT/CT experiments were carried out using a NanoSPECT/CT scanner (Mediso, Hungary). All procedures on animals were conducted in accordance with UK and Home Office regulations and the Guidance for the Operation of Animals (Scientific Procedures) Act (1986).

### 2.2 Chemical synthesis

Ethylene diamine (**1**) and 3,6-dioxaoctane-1,8-diamine (**2**) were converted into the mono-Boc compounds *tert*-butyl 2-aminoethylcarbamate (**4**) and *tert*-butyl 8-amino-3,6-dioxaoctylcarbamate (**5**) as previously described [34,35]. 3,6,9,12,15-Pentaoxaheptadecane-1,17-diamine (**3**) was prepared from hexaethylene glycol as previously reported [36,37]. 4-(2-(*Tert*-butoxycarbonylamino)ethylamino)-4-oxobutanoic acid (**7**) [38], 3-bis[(*Z*)-octadec-9-enyloxy]propan-1-amine (**10**) [39], 4,7,10-tris-*tert*-butoxycarbonylmethyl-1,4,7,10-tetraaza-cyclododec-1-yl)-acetic acid **14** [40], *N*-(2-(2-(2-(2-hydroxyethoxy)ethoxy)ethoxy)ethyl)-*N*,*N*-dimethyl-2,3-bis((Z)-octadec-9-enyloxy)propan-1-aminium (DODEG4) [10] and *N*-[1-(2,3-dioleyloxy)propyl])-*N*,*N*,*N*-trimethylammonium chloride (DOTMA) [39,41] were all prepared as previously described.

***tert*-Butyl 17-amino-3,6,9,12,15-pentaoxaheptadecylcarbamate** (**6**) The reaction was carried out under anhydrous conditions. To 3,6,9,12,15-pentaoxaheptadecane-1,17-diamine **3** (1.20 g, 4.28 mmol) in dichloromethane (100 mL) at 0 °C was added di-*tert*-butyl dicarbonate (0.470 g, 2.15 mmol). The solution was stirred at room temperature for 18 h, then the solvent removed *in vacuo* and the crude material purified via silica column chromatography (gradient 10–50% MeOH in CH_2_Cl_2_) to give **6** (comparable by NMR to the previous report [42]) as a colourless oil (0.594 g, 73%). R_f_ 0.40 (20% MeOH in CH_2_Cl_2_); ^1^H NMR (500 MHz; CDCl_3_) *δ* 1.42 (s, 9H), 2.72 (br s, 3H), 2.89 (t, *J* = 4.9 Hz, 2H), 3.30 (m, 2H), 3.53–3.65 (m, 20H); ^13^C NMR (125 MHz; CDCl_3_) *δ* 28.5, 41.7, 53.5, 70.2–70.6 (signals superimposed), 72.3, 79.2, 156.2; *m/z* (ES+) 381 (MH^+^, 100%).

**2,2-Dimethyl-4,15-dioxo-3,8,11-trioxa-5,14-diazaoctadecan-18-oic acid, triethylamine salt (8.triethylamine salt).** The reaction was carried out under anhydrous conditions. To *tert*-butyl 2-(2-(2-aminoethoxy)ethoxy)ethylcarbamate (**5**) (1.00 g, 4.03 mmol) in dichloromethane (50 mL) was added triethylamine (1.12 mL, 8.04 mmol) and succinic anhydride (0.443 g, 4.43 mmol). The reaction was stirred at room temperature for 3 h, then a further 0.4 equiv of succinic anhydride was added and the reaction stirred for 18 h. The solvent was removed under reduced pressure to yield a dark purple crude oil, which was purified *via* flash silica chromatography (gradient 5% MeOH and 1% triethylamine in CH_2_Cl_2_, to 10% then 20% MeOH in CH_2_Cl_2_) to give the triethylamine salt of **8** (comparable by NMR to the free acid [43]) (1.63 g, 90%) as a colourless oil. R_f_ 0.52 (10% MeOH in CH_2_Cl_2_); ^1^H NMR (500 MHz; CDCl_3_) *δ* 1.14 (t, *J* = 7.3 Hz, 9H), 1.35 (s, 9H), 2.38–2.46 (m, 4H), 2.89 (q, *J* = 7.3 Hz, 6H), 3.22 (m, 2H), 3.33 (m, 2H), 3.46 (m, 4H), 3.52 (m, 4H), 5.26 (br s, 1H), 7.10 (br s, 1H); ^13^C NMR (125 MHz; CDCl_3_) *δ* 8.6, 28.2, 32.6, 32.8, 38.9, 40.2, 44.8, 69.8–70.0 (signals superimposed), 78.9, 156.0, 173.5, 178.4; *m/z* [HRMS ES+] found, 371.1801. C_15_H_28_N_2_O_7_Na requires 371.1794.

**1-Amino-Boc-19-oxo-3,6,9,12,15-pentaoxa-18-azadocosan-22-oic acid (9).** The reaction was carried out under anhydrous conditions. To compound **6** (572 mg, 1.50 mmol) in dichloromethane (50 mL) was added triethylamine (420 μL, 3.01 mmol) and succinic anhydride (301 mg, 3.01 mmol). The solution was stirred at room temperature for 5 h, then the solvent removed under reduced pressure. The crude material was purified via silica column chromatography (gradient 5–20% MeOH in CH_2_Cl_2_) to give **9** (415 mg, 58%) as an oil. R_f_ 0.33 (10% MeOH in CH_2_Cl_2_); *ν*_max_(CHCl_3_)/cm^−1^ 3332, 2873, 1784, 1708; ^1^H NMR (500 MHz; CDCl_3_) *δ* 1.44 (s, 9H), 2.51 (t, *J* = 6.2 Hz, 2H), 2.60 (m, 2H), 3.30 (m, 2H), 3.43 (m, 2H), 3.53–3.67 (m, 20H), 5.24 (br s, 1H), 7.05 (br s, 1H); ^13^C NMR (125 MHz; CDCl_3_) *δ* 28.6, 31.7, 31.8, 39.4, 40.4, 69.8–70.6 (signals superimposed), 79.4, 156.3, 173.6, 176.8; *m/z* [HRMS ES+] found, 503.2583. C_21_H_40_N_2_O_10_Na requires 503.2581.

***N*1-(2-aminoethyl)-*N*4-(2,3-bis((*Z*)-octadec-9-enyloxy)propyl)succinamide (11).** To the acid **7** (555 mg, 2.13 mmol) was added HBTU (971 mg, 2.56 mmol) in anhydrous dichloromethane (30 mL), diisopropylethylamine (DIPEA) (744 μL, 4.27 mmol), and amine **10** (1.26 g, 2.13 mmol). The solution was stirred at room temperature for 4 h, then the solvent removed under reduced pressure and the crude product purified *via* silica column chromatography (5% MeOH in CH_2_Cl_2_) to yield the Boc-protected intermediate, *tert*-butyl 2-(4-(2,3-bis((*Z*)-octadec-9-enyloxy)propylamino)-4-oxobutanamido)ethyl carbamate (998 mg, 79%) as an light orange oil. R_f_ 0.57 (15% MeOH in CH_2_Cl_2_); ^1^H NMR (600 MHz; CDCl_3_) *δ* 0.87 (t, *J* = 7.0 Hz, 6H), 1.26 (m, 44H), 1.43 (s, 9H), 1.54 (m, 4H), 1.94–2.02 (8H, m, C*H*_2_CH = CHC*H*_2_), 2.47–2.53 (m, 4H), 3.24–3.35 (m, 4H), 3.37–3.57 (m, 9H), 5.04 (br t, 1H), 5.33 (m, 4H), 6.21 (br t, 1H), 6.52 (br t, 1H); ^13^C NMR (150 MHz; CDCl_3_) *δ* 14.3, 22.8, 26.2, 27.3, 28.8, 29.2–29.9 (signals superimposed), 30.2, 31.8, 32.0, 32.7, 38.7, 40.3, 40.6, 70.4, 71.5, 71.9, 76.6, 79.7, 129.9, 130.5, 156.8, 172.6, 174.5; *m/z* (ES+) 835 (MH^+^, 90%). The Boc group was then deprotected with 1:1 CH_2_Cl_2_:TFA (10 mL) for 3 h at room temperature. The solvents were removed *in vacuo* and the product dried under vacuo to give **11** (quantitative yield) as a light orange oil. R_f_ 0.39 (15% MeOH in CH_2_Cl_2_); *ν*_max_ (neat)/cm^−1^ 3289, 2924, 2853, 1647; ^1^H NMR (500 MHz; CDCl_3_) *δ* 0.87 (t, *J* = 7.0 Hz, 6H), 1.26 (m, 44H), 1.55 (m, 4H), 2.00 (m, 8H), 2.50 (m, 2H), 2.56 (m, 2H), 3.26 (m, 1H), 3.37–3.56 (m, 12H), 5.34 (m, 4H), 6.18 (br t, 1H), 6.90 (br t, 1H), 8.26 (br t, 2H); ^13^C NMR (150 MHz; CDCl_3_) *δ* 14.2, 22.8, 26.2, 27.3, 29.4–29.9 (signals superimposed), 30.1, 31.1, 31.6, 31.7, 32.0, 32.7, 39.0, 40.8, 41.1, 70.3, 71.5, 72.0, 76.4, 129.9, 130.1, 172.3, 174.4; *m/z* [HRMS ES+] found, 734.6710. C_45_H_88_N_3_O_4_ requires 734.6775.

***N*1-(2-(2-(2-aminoethoxy)ethoxy)ethyl)-*N*4-(2,3-bis((*Z*)-octadec-9-enyloxy)propyl) succinamide (12)** The reaction was carried out under anhydrous conditions. To the triethylamine salt of acid **8** (438 mg, 0.975 mmol) in dichloromethane (50 mL) was added HBTU (716 mg, 1.89 mmol) DIPEA (438 μL, 2.52 mmol) and amine **10** (496 mg, 0.840 mmol). The solution was stirred at room temperature for 18 h. The crude oil was dry loaded onto a silica column and purified *via* flash chromatography (0, 1%, 2%, then 5% MeOH in CH_2_Cl_2_) to yield the Boc-protected intermediate *tert*-butyl (*Z*)-16-((*Z*)-octadec-9-enyloxy)-10,13-dioxo-3,6,18-trioxa-9,14-diazahexatriacont-27-enyl carbamate (696 mg, 77%) as a colourless oil. R_f_ 0.54 (10% MeOH in CH_2_Cl_2_); ^1^H NMR (600 MHz; CDCl_3_) *δ* 0.87 (t, *J* = 6.9 Hz, 6H), 1.26–1.32 (m, 44H), 1.44 (s, 9H), 1.55 (m, 4H), 1.94–2.02 (m, 8H), 2.51 (m, 4H), 3.27–3.74 (m, 21H), 5.14 (br s, 1H), 5.30–5.36 (m, 4H), 6.15 (br s, 1H), 6.45 (br s, 1H); ^13^C NMR (150 MHz; CDCl_3_) *δ* 14.3, 22.8, 26.2, 27.3, 28.5, 29.3–29.9 (signals superimposed), 30.2, 32.0, 32.7, 39.4, 40.4, 41.0, 69.9, 70.3, 70.4 (signals superimposed), 71.5, 71.9, 79.4, 129.9, 130.4, 156.2, 172.1; *m/z* (ES+) 823 ([MH^+^-Boc], 100%). The Boc group was then deprotected with 1:1 CH_2_Cl_2_:TFA (5 mL) for 3 h at room temperature. The solvents were removed *in vacuo* and the product purified *via* silica column chromatography (gradient 5–15% MeOH in CH_2_Cl_2_) to give **12** in quantitative yield as a colourless oil. R_f_ 0.20 (10% MeOH in CH_2_Cl_2_); *ν*_max_(CHCl_3_)/cm^−1^ 3292, 2926, 2854, 1669; ^1^H NMR (600 MHz; CDCl_3_) *δ* 0.86 (t, *J* = 6.9 Hz, 6H), 1.24–1.28 (m, 44H), 1.54 (m, 4H), 1.94–2.0 (m, 8H), 2.52 (m, 4H), 3.14 (m, 2H), 3.23 (m, 1H), 3.36–3.58 (m, 10H), 3.63 (m, 4H), 3.66 (m, 2H), 3.75 (m, 2H), 5.30–5.39 (m, 4H), 6.56 (br s,1H), 7.83 (br s, 1H); ^13^C NMR (150 MHz; CDCl_3_) *δ* 14.2, 22.8, 25.1, 26.2, 27.4, 29.3–29.9 (signals superimposed), 30.1, 30.6, 30.9, 32.0, 32.7, 33.8, 39.5, 39.8, 41.1, 66.9, 69.8, 69.9, 70.4, 71.3, 71.9, 76.6, 129.9, 130.0, 173.5; *m/z* [HRMS ES+] found, 822.7242. C_47_H_97_N_3_O_6_Na requires 822.7275.

***N*1-(17-amino-3,6,9,12,15-pentaoxaheptadecyl)-*N*4-(2,3-bis((Z)-octadec-9-enyloxy) propyl) succinamide (13).** The reaction was carried out under anhydrous conditions. To the acid **9** (415 mg, 0.864 mmol) in dichloromethane (30 mL) was added HBTU (394 mg, 1.04 mmol), DIPEA (301 mL, 1.73 mmol) and amine **10** (512 mg, 0.865 mmol) in dichloromethane (5 mL). The solution was stirred at room temperature for 18 h, then the solvent removed under reduced pressure and the crude product purified *via* silica column chromatography (5% MeOH in CH_2_Cl_2_) to yield the Boc-protected intermediate, *tert*-butyl (*Z*)-25-((*Z*)-octadec-9-enyloxy)-19,22-dioxo-3,6,9,12,15,27-hexaoxa-18,23-diazapenta-tetra-cont-36-enylcarbamate (768 mg, 93%) as a light yellow oil. R_f_ 0.71 (10% MeOH in CH_2_Cl_2_); ^1^H NMR (600 MHz; CDCl_3_) *δ* 0.86 (t, *J* = 7.0 Hz, 6H), 1.23–1.31 (m, 44H), 1.45 (s, 9H), 1.53 (m, 4H), 1.94–2.01 (m, 8H), 2.51 (m, 4H), 3.17–3.72 (m, 35H), 5.29 (br s, 1H), 5.32–5.36 (m, 4H), 6.33 (br s, 1H), 6.73 (br s, 1H); ^13^C NMR (150 MHz; CDCl_3_) *δ* 14.3, 22.8, 26.2, 27.3, 28.5, 29.4–29.9 (signals superimposed), 30.2, 31.7, 31.8, 32.0, 32.7, 39.3, 40.3, 41.0, 43.7, 70.1–70.4 (signals superimposed), 71.5, 71.9, 79.4, 129.9, 130.4, 156.6, 172.4, 172.9; *m/z* (ES+) 1055 (MH^+^, 40%). The Boc group was then deprotected with 1:1 CH_2_Cl_2_:TFA (10 mL) for 3 h at room temperature. The solvents were removed *in vacuo* and the product purified *via* silica column chromatography (gradient 5–15% MeOH in CH_2_Cl_2_) to give **13** in quantitative yield as a light yellow oil. R_f_ 0.44 (10% MeOH in CH_2_Cl_2_); *ν*_max_(neat)/cm^−1^ 2925, 2855, 1778; ^1^H NMR (600 MHz; CDCl_3_) *δ* 0.86 (t, *J* = 7.0 Hz, 6H), 1.23–1.30 (m, 44H), 1.53 (m, 4H), 1.94–2.00 (m, 8H), 2.48 (m, 4H), 2.52 (m, 4H), 3.24 (m, 1H), 3.39–3.63 (m, 32H), 5.32–5.36 (m, 4H), 6.39 (br s, 1H), 7.01 (br s, 1H), 8.39 (br s, 2H); ^13^C NMR (150 MHz; CDCl_3_) *δ* 14.2, 22.8, 26.2, 27.3, 29.3–29.9 (signals superimposed), 30.1, 31.5, 31.8, 32.0, 32.7, 37.6, 39.4, 39.9, 40.9, 69.0, 69.9, 70.1, 70.4–70.6 (signals superimposed), 71.5, 71.9, 76.8, 129.9, 130.0, 172.3, 172.4; *m/z* [HRMS ES+] found, 954.8071. C_55_H_108_N_3_O_9_ requires 954.8086.

**2,2,2-(10-((*Z*)-13-((*Z*)-octadec-9-enyloxy)-2,7,10-trioxo-15-oxa-3,6,11-triazatritriacont-24-enyl)-1,4,7,10-tetraazacyclododecane-1,4,7-triyl)triethanoic acid (15).** The reaction was carried out under anhydrous conditions. To the DOTA derivative **14** (15.6 mg, 27.3 μmol), HBTU (15.5 mg, 42 μmol) and DIPEA (10 μL, 57.5 μmol) in dichloromethane (5 mL) was added amine **11** (20 mg, 27.3 μmol). The reaction was stirred at room temperature for 4 h, after this time the solvent was removed under reduced pressure and the crude product purified via silica column chromatography (0–2% MeOH in CH_2_Cl_2_) to give the protected intermediate, 2,2,2-(10-((*Z*)-13-((*Z*)-octadec-9-enyloxy)-2,7,10-trioxo-15-oxa-3,6,11-triazatritriacont-24-enyl)-1,4,7,10-tetraazacyclododecane-1,4,7-triyl)triethanoic acid *tri-tert-*butyl ester (10 mg, 28%) as a colourless oil. ^1^H NMR (600 MHz; CDCl_3_) *δ* 0.87 (t, *J* = 7.0 Hz, 6H), 1.26–1.30 (m, 44H), 1.45 (m, 27H), 1.55 (m, 4H), 2.01 (m, 8H), 2.51 (m, 4H), 3.28–3.56 (m, 21H), 5.36 (m, 4H), 6.35 (br t, *J* = 5.5 Hz, 1H), 6.48 (br t, *J* = 5.6 Hz, 1H), 6.82 (br t, *J* = 5.4 Hz, 1H), note – signals masked under line broadening 2–3 ppm; ^13^C NMR (150 MHz; CDCl_3_) *δ* 14.3, 22.8, 26.2, 27.4, 28.0 (signals superimposed), 29.3–29.9 (signals superimposed), 30.2, 32.0, 32.2, 32.8, 39.3, 39.6, 40.8, 48.4 (br), 52.7 (br), 55.8 (signals superimposed), 70.4, 71.5, 71.8, 82.1, 130.0, 130.5, 172.1, 172.5, 173.3; *m/z* (ES+) 1288 (MH^+^, 30%), 645 ([MH_2_]^2+^/2, 90%). The tri-*tert*-butyl ester was then deprotected in 1:1 CH_2_Cl_2_:TFA (5 mL) for 3 h at room temperature. The solvents were removed *in vacuo* and the product dried under vacuo to give **15** (quantitative yield) as a colourless oil. R_f_ 0.63 (15% MeOH in CH_2_Cl_2_); *ν*_max_(CHCl_3_)/cm^−1^ 2924, 2854, 1652; ^1^H NMR (600 MHz; CD_3_OD) *δ* 0.90 (t, *J* = 7.0 Hz, 6H), 1.29–1.38 (m, 44H), 1.51 (m, 4H), 1.98–2.05 (m, 8H), 2.51 (m, 4H), 3.19–3.73 (m, 21H), 5.35 (m, 4H), note – signals masked by line broadening 3.2–3.7 ppm; ^13^C NMR (150 MHz; CD_3_OD) *δ* 14.5, 23.8, 27.3, 28.2, 28.5, 30.2–30.9 (signals superimposed), 31.2, 33.1, 33.7, 34.7, 39.6, 40.2, 41.7, 43.8, 55.8, 71.3, 72.4, 72.6, 78.6, 130.8, 131.5, 161.3, 174.8, 175.2; *m/z* [HRMS ES+] found, 1120.8534. C_61_H_114_N_7_O_11_ requires 1120.8576.

**2,2,2-10-((*Z*)-19-((*Z*)-octadec-9-enyloxy)-2,13,16-trioxo-6,9,21-trioxa-3,12,17-triaza nona triacont-30-enyl)-1,4,7,10-tetraazacyclododecane-1,4,7-triyl) triethanoic acid (16).** The reaction was carried out under anhydrous conditions. To the DOTA derivative **14** (60 mg, 0.105 mmol) in dichloromethane (10 mL), was added HBTU (40 mg, 0.105 mmol) and DIPEA (37 μL, 0.212 mmol) and amine **12** (86 mg, 0.105 mmol). The reaction was stirred at room temperature for 4 h, the solvent removed under reduced pressure and the crude product purified via silica column chromatography (6% MeOH in CH_2_Cl_2_) to afford the protected intermediate, 2,2,2-10-((*Z*)-19-((*Z*)-octadec-9-enyloxy)-2,13,16-trioxo-6,9,21-trioxa-3,12,17-triazanonatriacont-30-enyl)-1,4,7,10-tetraazacyclododecane-1,4,7-triyl) triethanoic acid *tri-tert*-butyl ester (93 mg, 65%) as a colourless oil. ^1^H NMR (500 MHz; CDCl_3_) *δ* 0.87 (t, *J* = 7.2 Hz, 6H), 1.25 (m, 44H), 1.45 (m, 27H), 1.55 (m, 4H), 1.95–2.02 (m, 8H), 2.51 (m, 4H), 3.26–3.30 (m, 2H), 3.37–3.65 (m, 19H), 5.33–5.38 (m, 4H), 6.38 (br t, *J* = 5.6 Hz, 1H), 6.48 (br t, *J* = 5.9 Hz, 1H), 6.74 (br t, *J* = 5.6 Hz, 1H), note – signals masked under line broadening 2.0–3.2 ppm; ^13^C NMR (125 MHz; CDCl_3_) *δ* 14.3, 22.8, 26.2, 27.3, 28.0, 28.1, 29.3–29.9 (signals superimposed), 30.2, 31.8, 32.0, 32.8, 38.7, 39.3, 40.9, 68.9, 69.6, 69.8, 70.3, 70.4, 71.6, 71.9, 72.0, 82.0, 130.0, 130.5, 171.9, 172.4, 172.5, 172.6; *m/z* (ES+) 1377 (MH^+^, 100%). The tri-*tert*-butyl ester was then deprotected in 1:1 CH_2_Cl_2_:TFA (5 mL) for 3 h at room temperature. The solvents were removed *in vacuo* and the product dried under vacuo to give **16** (quantitative yield) as a colourless oil. R_f_ 0.81 (10% MeOH in CH_2_Cl_2_); *ν*_max_(CHCl_3_)/cm^−1^ 2926, 2854, 1724, 1663; ^1^H NMR (600 MHz, CD_3_OD) *δ* 0.90 (t, *J* = 7.0 Hz, 6H), 1.22 (m, 44H, m), 1.56 (m, 4H), 1.98–2.05 (m, 8H), 2.49 (s, 4H), 3.19–3.65 (m, 21H), 5.34–5.39 (m, 4H), note – some signals masked under line broadening 3.2–3.7 ppm; ^13^C NMR (125 MHz, CDCl_3_) *δ* 14.5, 23.8, 26.1, 27.3, 28.2, 30.2–30.9 (signals superimposed), 31.2, 32.1, 32.2, 33.0, 33.1, 33.7, 34.7, 38.9, 40.3, 41.6, 69.8, 70.4, 70.5, 71.2, 71.3, 72.4, 72.6, 78.6, 81.3, 130.8, 131.6, 174.7; *m/z* [HRMS ES+] found, 1208.8995. C_65_H_122_N_7_O_13_ requires 1208.9101.

**2,2,2-10-((*Z*)-28-octadec-9-enyloxy)-2,22,25-trioxo-6,9,12,15,18,30-hexaoxa-3,21,26-triaza octa tetracont-39-enyl)-1,4,7,10-tetraazacyclododecane-1,4,7-triyl)triethanoic acid (17).** The reaction was carried out under anhydrous conditions. To the DOTA derivative **14** (140 mg, 0.244 mmol) in dichloromethane (10 mL) was added HBTU (111 mg, 0.293 mmol), DIPEA (213 μL, 1.22 mmol) and amine **13** (280 mg, 0.294 mmol). The reaction was stirred at room temperature for 3 h, the solvent removed under reduced pressure and the crude product purified *via* silica column chromatography (gradient 5–10% MeOH in CH_2_Cl_2_) to yield the protected intermediate, 2,2,2-10-((*Z*)-28-octadec-9-enyloxy)-2,22,25-trioxo-6,9,12,15,18,30-hexaoxa-3,21,26-triazaoctatetracont-39-enyl)-1,4,7,10-tetraazacyclododecane-1,4,7-triyl)triethanoic acid *tri-tert*-butyl ester (199 mg, 54%) as a light yellow oil. ^1^H NMR (600 MHz; CDCl_3_) *δ* 0.87 (t, *J* = 6.9 Hz, 6H), 1.26 (m, 44H), 1.44 (s, 27H), 1.55 (m, 4H), 2.0 (8H, m, C*H*_2_CH]CHC*H*_2_), 2.52 (4H, m, OCC*H*_2_C*H*_2_CO), 3.20–3.65 (m, 35H), 5.34 (m, 4H), 6.42 (br s, 2H), 6.84 (br s, 1H), 6.98 (br s, 1H), note – signals masked under line broadening 2.0–3.2 ppm; ^13^C NMR (150 MHz; CDCl_3_) *δ* 14.3, 22.8, 26.2, 27.3, 27.9–28.1 (signals superimposed), 29.3–29.9 (signals superimposed), 30.2, 31.6, 31.9, 32.0, 32.7, 33.8, 39.2, 39.3, 40.9, 48.5 (br), 53.0 (br), 55.8 (br), 56.1, 56.8, 69.7, 70.0, 70.1, 70.2, 70.4, 71.5, 71.9, 81.8, 81.9, 82.3, 130.0, 130.5, 171.8, 172.4, 172.6, 173.2; *m/z* (ES+) 1476 ([MNa – ^t^Bu]^+^, 100%). The tri-*tert*-butyl ester was then deprotected in 1:1 CH_2_Cl_2_:TFA (5 mL) for 3 h at room temperature. The solvents were removed *in vacuo* and the product dried under vacuo to give **17** as a colourless oil (quantitative yield). R_f_ 0.56 (10% MeOH in CH_2_Cl_2_); *ν*_max_(CHCl_3_)/cm^−1^ 2926, 2856, 1736; ^1^H NMR (600 MHz; CD_3_OD) *δ* 0.89 (t, *J* = 6.9 Hz, 6H), 1.30 (m, 44H), 1.56 (m, 4H), 2.02 (m, 8H), 2.48 (m, 4H), 3.18–3.64 (m, 35H), 5.33 (m, 4H), note – some signals masked under line broadening 2.9–3.6 ppm; ^13^C NMR (150 MHz; CDCl_3_) *δ* 14.5, 23.8, 26.1, 27.3, 28.2, 28.5, 30.3–30.9 (signals superimposed), 31.2, 32.2, 33.1, 33.7, 34.7, 38.9, 40.4, 41.6, 70.5 71.1–71.6 (signals superimposed), 72.6, 78.6, 81.3, 130.8, 130.9, 162.9, 163.1, 167.5, 174.7, 174.9, 179.7; *m/z* [HRMS ES+] found, 1340.9716. C_71_H_134_N_7_O_16_ requires 1340.9887.

### 2.3 Liposome preparation and characterization

The Gd lipids **Gd-DEG1SL**, **Gd-DEG3SL** and **Gd-DEG6SL** were prepared [14] from **15**, **16** and **17**, respectively, as follows. The free acid – macrocyclic lipids (**15**, **16**, **17**) were each dissolved in 2 mL of distilled water, then 0.9 molar equivalents of GdCl_3_ were added. The solutions were heated at 90 °C overnight, then freeze dried to a powder and stored at −20 °C. Successful chelation was confirmed via Xylenol Orange assay [44] and MALDI MS (Supplementary Data, Figs. S1–S3).

MALDI MS data for **Gd-DEG1SL**, 1274, 1275, 1276, 1277, 1278 g mol^−1^

MALDI MS data for **Gd-DEG3SL**; 1361, 1362, 1363, 1364, 1365, 1366, 1367 g mol^−1^

MALDI MS data for **Gd-DEG6SL**; 1493, 1494, 1495, 1496, 1497, 1498, 1499 g mol^−1^

All lipid components were dissolved in either CHCl_3_ or methanol to a concentration of 1 mm. The lipids were mixed together in the appropriate molar ratios, shown in Table 1 (incorporating Gd-lipids **Gd-DEG1SL**, **Gd-DEG3SL** and **Gd-DEG6SL**) and Table 2 (incorporating the DOTA-lipids **DEG1SL** (**15**), **DEG3SL** (**16)** and **DEG6SL** (**17**) for subsequent labelling of the liposomes with ^64^Cu or ^111^In) and the solvents removed under reduced pressure to form a thin film. This film was further dried on a high vacuum line for 4 h then hydrated with sterilized water, diluted to the required concentration and stored at 4 °C overnight. After this period the sample was sonicated for 10 min and used immediately. For *in vitro* assays a bath sonicator was used due to the small sample volume. For *in vivo* injections a probe sonicator (Sonifier SLPe, Branson (USA)) was used. The liposomes were characterized by DLS and zeta potential (Tables 1 and 2) according to manufacturers recommendations. The samples were prepared by taking a 50 μL aliquot of the stock liposome solution, which had a total lipid concentration of 3.33 mm, this was then diluted in H_2_O to a final volume of 1 mL. Both DLS and zeta potential measurements were performed at 25 °C, in triplicate, using a clear 1 mL zeta potential cuvette.

### 2.4 Phantom studies

A range of concentrations (serial dilutions 1–0.0625 mm) of **Gd-DEG1SL**, **Gd-DEG3SL** and **Gd-DEG6SL** were used to calculate relaxivity of each contrast agent as liposome formulations **A** – **G**. The relaxivities of these formulations were compared to the relaxivity of Gd.DOTA (Dotarem^®^). Phantoms were imaged by MR using a fast spin-echo sequence with the following parameters to obtain images with increasing T_1_-weighting; 10 TR’ = 200–15,000 ms, TE = 17 ms, FOV = 40 × 40 cm, averages = 4: matrix size = 256 × 128: and a 1.0 mm thickness.

### 2.5 Radiolabeling with ^111^In

For Instant Thin Layer Chromatography (ITLC), the liposomes in water (20 μL) were buffered with 0.1 m ammonium acetate buffer, pH 6 (20 μL). ^111^InCl_3_ (20 μL, 16 MBq) was added and the reaction incubated at 37 °C for 90 min. A sample (10 μL) of each reaction was challenged with 50 mm EDTA (2 μL) for 5 min to bind any free or non-specifically bound ^111^In. Samples were then analysed by ITLC (Supplementary Data, Table S2). Controls, using water in place of liposomes, were treated as per the liposome samples.

For *in vivo* studies, the liposomes were prepared as above but with the following changes; to the liposome solution (in sterilised water, 300 μL) were added 0.1 M ammonium acetate buffer (pH 6.0, 300 μL) and ^111^InCl_3_ solution (200 μL). The sample was incubated at room temperature overnight, after which the liposomes were purified to remove free ^111^InCl_3_ by eluting through a PD10 size exclusion column (GE Healthcare) prior to injection [45]. The radioactivity of the ^111^InCl_3_ solution was around 45 MBq; in a typical example, from 41.5 MBq of ^111^InCl_3_ solution 22.12 MBq of radiolabelled liposomes were recovered after purification (radiochemical yield of 69.2% accounting for decay of ^111^In).

### 2.6 Radiolabelling with ^64^Cu

**[**^64^Cu]-CuCl_2_ was buffered with an equal volume of 1 m ammonium acetate, pH 6. Typically, the liposomes in water (10 μL) were buffered with 0.1 m ammonium acetate buffer, pH 6, (10 μL). Buffered ^64^Cu solution (10 μL, 3 MBq) was added and the reaction incubated at 37 °C for 30 min. A sample (10 μL) of each reaction was challenged with 50 mm EDTA (2 μL) for 5 min to bind any free or non-specifically bound ^64^Cu. Samples were then analysed by ITLC. Control, using water in place of liposome, was treated as per the liposome samples.

Analysis of the radiolabelled liposomes was carried out by ITLC using ITLC-SA (0.75 × 9 cm) with a 2 μL sample spotted at the origin, 1 cm from the bottom of the strip, and run for 8 cm in 0.1 m citrate buffer, pH 5. ITLC strips were scanned on a mini-TLC scanner and analysed with Laura software (LabLogic). For both ^111^In and ^64^Cu, analysis of the control reaction using 0.1 m ammonium acetate buffer alone with no liposome gave 100% of the radioactivity at the solvent front using this ITLC system (Supplementary Data, Tables S1 and S2).

### 2.7 In vitro studies

Human HeLa cervical cancer, OVCAR-3 ovarian cancer, and MDA-MB-231 breast cancer cells were grown in T175 flasks (Fisher Scientific, Loughborough, UK) in Dulbecco's modified Eagles medium (DMEM) (Invitrogen, Paisley, UK), supplemented with 10% heat inactivated fetal calf serum (GIBCO, Grand Island NY, USA) in an humidified incubator at 37 °C with 95% air and 5% CO_2_. Cells were grown to 100% confluence before being trypsinized, counted and then plated for *in vitro* experiments.

HeLa, OVCAR-3 and MDA-MB-231 cells were plated at 5 × 10^5^/well in 6 well plates (for microscope and FACS analysis) or 1 × 10^6^/flask in a T25 flask (for MRI) 24 h prior to adding liposomes. The cells were washed in phosphate buffered saline (PBS) (GIBCO, USA) and 2 mL of serum free or normal culture DMEM was added, followed by 100 μL of either 1, 0.5, 0.025 mm of liposome or water as control. Cells were incubated at 37 °C for 2 h (normal culture media) or 4 h (serum free media). The cells were washed using PBS and fluorescence imaged using an inverted Zeiss Axiovert S100 Microscope. After harvesting cells were counted using 4% Trypan Blue solution (Sigma–Aldrich, Dorset, UK) to assess the number of viable cells and then resuspended in 500 μL PBS for FACS scanning using a FACS Calibur (Becton Dickinson, Franklin Lakes NJ, USA) to assess cell uptake (1 × 10^5^ cells), or pelleted and resuspended in 1% agarose (Sigma, UK) (2 × 10^6^ cells) and scanned by MRI using the same set up and parameters as phantoms to assess *T*_1_ changes. (Supplementary Data Fig. S5, Table S3).

### 2.8 MRI data analysis (relaxivity and *T*_1_)

Images were analysed using ImageJ software (National Institutes of Health, USA [46] with a region of interest (ROI) drawn to encompass as much of the sample as possible. The mean signal intensities of the ROI's at different TR values were measured and used to calculate the MR relaxation time *T*_1_ using Graphpad Prism (GraphPad, San Diego, USA). The longitudinal relaxivity *r*_1_ was determined from a linear fit of 1/T_1_ as a function of Gd^3+^ concentration (Table 3). Mean *T*_1_ values for labelled cell pellets were derived and a two-tailed unpaired *t*-test assuming equal variances performed to determine significant difference, with a 5% level of statistical significance. The percentage change in *T*_1_ was also calculated for these samples.

### 2.9 In vivo SPECT/CT acquisition and image analysis

Eight male NOD Scid gamma (NSG) mice (6–8 weeks old) (*n* = 2 per liposome **L**, **N**, **O** or **P**) were anaesthetized with an isoflurane/O_2_ mix and a tail vein cannulated for the delivery of approximately 10 MBq of ^111^In labelled liposomes in approximately 300–400 μl sterile saline. SPECT/CT scans were acquired for the whole body with mice placed in the prone position immediately post injection and again at 3, 6 and 24 h using a NanoSPECT/CT scanner (Mediso, Hungary). CT images were acquired using a 45 kVP X-ray source, 500 ms exposure time in 180 projections, a pitch of 0.5 with an acquisition time of 11 min. CT was imaged prior to SPECT, which was acquired using an exposure time of 1200 s, obtained over 24 projections (50 s per projection), a 4-head scanner with 4 × 9 1 mm pinhole apertures in helical scan mode with a total acquisition time of 36 min. CT images were reconstructed in a 352 × 352 matrix using proprietary Bioscan InVivSCope (Bioscan, USA) software, whereas SPECT images were reconstructed in a 256 × 256 matrix using HiSPECT (ScivisGmbH, Bioscan). Images were fused and analysed using InVivoScope (Version 1.44, Bioscan). 3D ROI's were created for heart, lung, liver, spleen, kidneys and bladder at each time point using InviCRO 3D and the counts decay corrected and compared to the total injected at the 40 min time point for each mouse to assess biodistribution and clearance. The bladder was removed from the 40 min and 3 h images, allowing the images to be scaled so that they were comparable to each other.

## 3 Results

### 3.1 Synthesis of DOTA chelating lipids

**Scheme 1:**
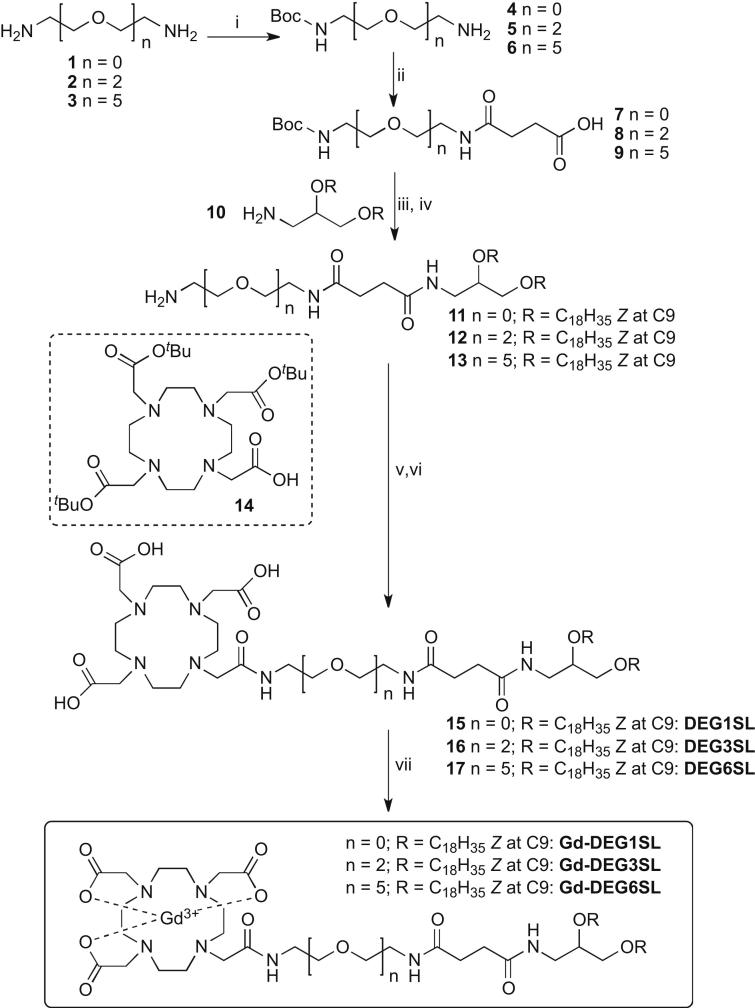
*Synthesis of DOTA-lipids and Gd-DOTA lipids*. Reagents and conditions: (i) (Boc)_2_O, CH_2_Cl_2_, **4** 33%, **5** 84%, **6** 73%; (ii) NEt_3_, succinic anhydride, **7** 55%, **8** 90%, **9** 58%; (iii) amine 10 and either HBTU and DIPEA, or DCC, CH_2_Cl_2_; (iv) TFA/CH_2_Cl_2_, **11** 79%, **12** 77%, **13** 93% over 2 steps; (v) **14**, HBTU, DIPEA, CH_2_Cl_2_; (vi) TFA/CH_2_Cl_2_, **15** 28%, **16** 65%, **17** 54% over 2 steps; (vii) GdCl_3_, H_2_O.

As we aimed to develop a multimodal liposomal formulation which would also have an evenly coated *n*-EG shielding functionality, DOTA conjugates were prepared incorporating different length short ethylene glycol spacers between the C18 unsaturated lipophilic chains and the DOTA chelator. To enhance ready assembly and further protrusion of the DOTA chelate on the exterior of the nanoparticle a succinimide spacer was also incorporated. Synthesis of the DOTA-conjugates was carried out as shown in Scheme 1, incorporating an aminodiol skeleton with two unsaturated oleyl chains via ether links, also present in the shielding cationic lipid DODEG4 (Fig. 1) to be used in this study. Accordingly, diamines **1**, **2**, and **3** [36,37] were readily mono-Boc protected to give **4**, **5** [34,35] and **6** [42] respectively. Addition of succinic anhydride gave **7** [38], **8** and **9** in good yield. 3-Bis[(*Z*)-octadec-9-enyloxy]propan-1-amine (**10**) was synthesized as previously reported [39] and coupled to **7**–**9** using standard HBTU or DCC chemistry, then directly deprotected to give **11**, **12** and **13**. These were then conjugated to the macrocycle 4,7,10-tris-*tert*-butoxycarbonylmethyl-1,4,7,10-tetraaza-cyclododec-1-yl)-acetic acid **14**, synthesized using reported procedures [40] again using standard HBTU reaction conditions. The *tert*-butyl ester products were deprotected under acidic conditions to give **15**, **16** and **17**, the key DOTA lipid chelates for incorporation of Gd^3+^ or other metal ions.

In order to investigate the use of these liposomes in MR imaging applications, chelation of Gd^3+^ with **15**, **16** and **17** was first carried out. The key lipid chelates were dissolved in distilled water and stirred overnight at 90 °C with GdCl_3_; 0.9 molar equivalents were used to ensure that all of the Gd^3+^ was chelated. The solution was lyophilized overnight and the free Gd^3+^ content quantitatively analysed via the Xylenol Orange assay [44], confirming negligible free Gd^3+^. MALDI MS analysis of the lipid salts gave the desired masses, corresponding to lipids chelated to each of the seven stable Gd isotopes (Supplementary Data, Figs. S1–S3).

### 3.2 Formulation of liposomes for use as MR contrast agents

To investigate the application of liposomes as multimodal agents, co-formulation of the Gd^3+^-lipids with multiple lipid components was necessary. Introduction of a fluorophore allows tracking and quantification of the nanoparticle via optical means, therefore the commercially available fluorophore-labelled lipid FL-DHPE was employed. The fusogenic lipid DOPE was also used in the formulations, along with the cationic lipids DOTMA and DODEG4.

In order to investigate the effects of the length of the linker of the Gd-lipids on MR relaxivity, and PEG shielding of the liposome on cellular uptake, a series of liposomes **A**–**G** were formulated (Table 1). Liposomes **A**–**C** and **E–G** were formulated with 9% DOPE, which is believed to promote liposome fusion with the endosomal membrane and mediate endosomal escape of the payload once the liposome has been internalized [47], and 1 mol% FL-DHPE was added to all formulations. Gd-lipids **Gd-DEG1SL** (liposomes **A** and **E**), **Gd-DEG3SL** (liposomes **B**, **D** and **F**) and **Gd-DEG6SL** (liposomes **C** and **G**) were added at 30 mol% to enable the liposomes to be imaged using MR. In order to shield the liposomes from interactions with other biomolecules and promote stability under physiological conditions, liposomes **A**, **B** and **C** were formulated with 50 mol% DODEG4, a non-cleavable tetraethylene glycol lipid that we have developed previously [10]. At this high percentage it was reasoned that the liposome should be coated with an even coverage of short *n*-ethylene glycol units. For comparison, liposome **D** was formulated with the stealth lipid DSPE-PEG2000 at 7 mol% (partially replacing the DOPE in the other formulations) which has previously been used at similar mol% to produce sterically shielded liposomes [48]. Non-shielded liposomes **E**, **F** and **G** were formulated without incorporating either DODEG4 or DSPE-PEG2000. For all formulations, an appropriate amount of the cationic lipid DOTMA was also added, to give liposomes with a total of 60% of positively charged lipids incorporated.

Liposomes were formed using the thin-film sonication method [11], to give uniform liposomes of low polydispersity and hydrodynamic sizes ranging between 90 and 250 nm (Table 1). The non-shielded liposomes **E**, **F** and **G** were significantly larger, perhaps due to aggregation. The zeta potential of liposomes **A**–**G** was also measured (Table 1); despite the differences in PEG shielding, all liposomes had similar, positive surface charges.

### 3.3 MR analysis of liposomal agents

Phantoms of serial dilutions from a 1 mm stock solution of Gd labelled liposomes **E**, **F** and **G**, formulated without PEG shielding, showed the relaxivity of the solutions to be 3.05, 2.37 and 1.78 mm^−1^ s^−1^ respectively (Table 3). These values are comparable to the relaxivity of non-conjugated Gd.DOTA (Dotarem), which was measured as 2.68 mm^−1^ s^−1^. This suggests that although the functionalization of one arm of the DOTA macrocycle with the lipid moiety does not adversely affect the relaxivity of the macrocyclic compound. The fact that all the relaxivities are slightly different suggests that the length of the *n*-EG linker does in fact have some influence on the relaxivity.

Relaxivities of liposome formulations including the DODEG4 lipid were also measured, in order to investigate the effect of the interaction between this lipid with a short (*n* = 4) *n*-EG group attached, and the various *n*-EG linker-lengths positioned between the lipid head group and the DOTA chelator. Liposome formulations **A** (containing **Gd-DEG1SL**) and **B** (containing **Gd-DEG3SL**) with 50 mol% DODEG4 show a very slight reduction in *r*_1_ compared to their non-PEGylated counterparts **E** and **F** respectively (<5%). In contrast, a slight increase in relaxivity is seen for **C** (containing **Gd-DEG6SL**), compared to the non-pegylated liposomal formulation **G**. This indicated that the short *n*-EG did not have a substantial further effect on relaxivity when compared to the non pegylated liposomes.

The relaxivity of liposome **D**, formulated with the lipid **Gd-DEG3SL** (with a linker of intermediate length) and with the previously reported stealth lipid DSPE-PEG2000 was also measured. In this case, the relaxivity of liposome **D** is comparable to the both the other **Gd-DEG3SL** preparations if slightly reduced in comparison to that of liposome **B** (with a shallow *n*-EG coating provided by DODEG4) and liposome **F** (no EG coating). This suggests that the *n*-EG linker on the DOTA-lipid has a greater influence on the relaxivity than the *n*-EG or PEG shielding provided by the other lipids. Nonetheless, both the *n*-EG and non-*n*-EG liposomes reduced *T*_1_ by approx. 85–90% relative to water control and are therefore can all be considered competent MRI contrast agents. It should be noted that we are comparing the relaxivities relative to the gadolinium concentration to assess the effect of the formulation on relaxivity. However, the actual relaxivity for each liposome is much greater: each liposome will incorporate multiple Gd-lipids, and thus the Gd payload per nanoparticle [19] is increased.

### 3.4 Cellular uptake

**Fig. 2:**
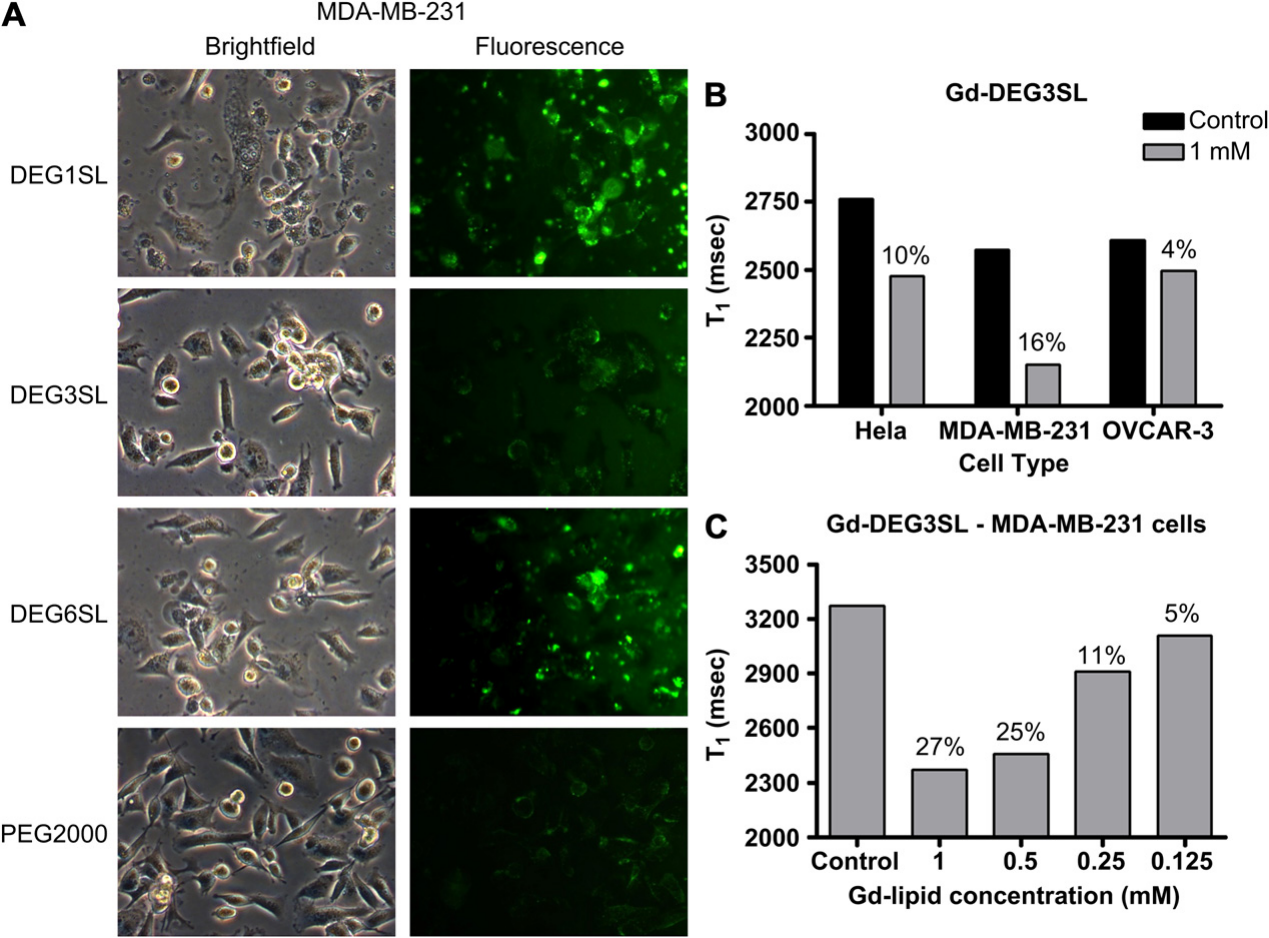
A) Brightfield and corresponding Fl-DHPE fluorescence images for MDA-MB-231 cells incubated with DODEG4 liposomes **A-C** formulated with **DEG1SL**, **DEG3SL** and **DEG6SL** respectively, compared to the control PEG2000 liposome **D** formulated with **DEG3SL** (X100 magnification). B) Changes in T_1_ relative to the degree of uptake of **Gd-DEG3SL** (liposome **B**) for three different cell types HeLa, MDA-MB-231, and OVCAR-3 compared to control cells. C) Dose dependent changes in T_1_ for MDA-MB-231 cells incubated with **Gd-DEG3SL** (liposome **B**).

Inclusion of the fluorescein lipid allowed confirmation and quantitative analysis of cellular liposomal uptake using FACS (Supplementary Data, Fig. S4 and Table S3) and fluorescence microscopy (Fig. 2A, also Supplementary Data, Figs. S5 and S6) and cell viability was also assessed by cell counting with Trypan Blue (Supplementary Data, Fig. S7). All cell lines investigated (HeLa, MDA-MB-231 and OVCAR-3) showed varying degrees of uptake for both the *n*-EG-shielded liposomes **A**, **B** and **C** (Fig. 2) as well as the non-shielded liposomes **E**, **F** and **G** (Supplementary Data, Fig. S5) by fluorescence microscopy. It was clear that the OVCAR-3 cells took up the least of all three cell lines based on the MRI T_1_ relaxation, with the MDA-MB-231 cells having the highest uptake (Fig. 2B). However, the OVCAR-3 cells also appeared to be more sensitive to liposome uptake, in general exhibiting lower cell viability after liposome incubation (Supplementary Data, Fig. S7). In contrast, cells labelled with liposome **D** (containing PEG2000) were shown to have less fluorescence. This was the only liposome preparation to present as a halo around the cell, indicative of being electrostatically bound to the cell surface rather than internalised (Fig. 2A). FACS (Supplementary Data, Table S3) confirmed that cells incubated with liposomes **A**-**G** were labelled (all liposomes label over 94% of cells) but that cells labelled with liposome **D,** although exhibiting the same percentage labelling, had a reduced fluorescence shift in the FACS plot (Supplementary Data, Fig. S4). FACS also indicated that there were no differences in the labelling of cells with **E**-**F** compared to **A**-**C** and also no differences in labelling of samples labelled in serum free compared to normal culture media (Supplementary Data, Table S3). However, fluorescent images suggested that the cells incubated with non-shielded liposomes (Supplementary Data, Fig. S5) and the cells incubated with shielded liposomes **B** and **C** in normal media (Supplementary Data, Fig. S6) showed an increase in fluorescence.

Out of liposomes **A–C**, liposome **B** (formulated with **DEG3SL**) was the best tolerated at higher doses (Supplementary Data, Fig. S7: 1 mm and 0.5 mm) but showed the lowest uptake by fluorescence. Liposomes **A** and **C** had the highest uptake; however, highly fluorescent cells were seen to round at high doses losing adherence and reducing cell viability at high concentration (1 mm and 0.5 mm) in both cases (Supplementary Data, Fig. S7). *n*-EG-shielded liposomes **A–C** showed less cell rounding compared to the non-shielded equivalents **E**–**F**, suggesting DODEG4 inhibits excessive uptake. Of the non-shielded liposomes, fluorescence images of **F** (also formulated with **DEG3SL**, as in **B**) had the highest fluorescence, but liposomes **E** and **G** labelled far fewer cells (Supplementary Data, Fig. S5). This may be indicative of high uptake rounding and cell loss prior to imaging, and the fluorescence seen for **E** and **G** may therefore be an underestimation. Uptake in cells in normal culture media also resulted in more highly fluorescent cells compared to serum free media, indicating greater uptake, but again accompanied by increased cell rounding (Supplementary Data, Fig. S6). Finally, liposome **D**, formulated including DSPE-PEG2000, led to the least reduction in cell viability, probably because these liposomes do not appear to be internalised.

MRI results (Fig. 2C) suggested that liposome **B** (the best tolerated by the cell lines) gave dose dependent *T*_1_ reduction with maximal 27% decrease in *T*_1_ compared to unlabelled control cells. Due to the increased uptake of liposomes **A** and **C**, leading to highly fluorescent cells with high concentrations of liposomes losing adherence and reducing cell viability, liposomes **A** and **C** were not used for MRI analysis. It could be presumed that the increase in concentration of Gd-liposome within the cell would give a greater effect on *T*_1_ for these liposome formations. However, due to differences in their relaxativity and a reduction in water interaction due to internalization within the cell any differences in *T*_1_ cannot be predicted.

### 3.5 Liposome radiolabelling with Cu-64 and In-111

In order to extend the imaging repertoire of this delivery system to include PET and SPECT, chelation of the appropriate radionuclides to give ^64^Cu or ^111^In-labelled liposomes was investigated. In order to optimise the conditions for efficient complexation, DOTA-lipids **DEG3SL** (**16**) and **DEG6SL** (**17**) were initially labelled, unformulated, with ^64^Cu; labelling efficiencies of 96% (**16**) and 77% (**17**) were obtained. (Supplementary Data, Table S1).

In the preceding *in vitro* MRI experiments, Gd-liposomes formulated from **Gd-DEG3SL** or **Gd-DEG6SL** were observed to have low and high uptake and internalization properties respectively. Liposomes containing these DOTA-lipids were used for post-formulation radiolabeling to give either ^111^In or ^64^Cu-labelled liposomes. Liposomes **H**–**M** and **O**, composed of 30 mol% of the uncomplexed DOTA-lipids **DEG3SL** or **DEG6SL**, were formulated with and without DODEG4 (Table 2), ensuring, as in all previous cases, that 60% of the lipids carry a positive charge, thus keeping the net charge on the liposome consistent. For comparison, liposome **N** was formulated with 7 mol% of DSPE-PEG2000 and 30 mol% **DEG3SL**, and liposome **P** was formulated with 7 mol% of DSPE-PEG2000 and 30 mol% **DEG6SL**, again adjusting the percentages of DOTMA and DOPE to keep the net positive charge consistent. Liposomes **Q** and **R**, with and without DODEG4, were formulated without DOTA-lipids as controls.

Liposomes **H**, **I**, **J**, **K**, **Q** and **R** (at a concentration of 1 mM with respect to the DOTA-lipid) were labelled with 3 MBq of ^64^Cu (Table 2), and liposomes **L**, **M**, **N, O and P** were labelled with 16 MBq of ^111^In. After 1 h, EDTA was added to sequester any unbound ions. The sample was analysed using TLC and plates were visualised with a gamma detector. The labelling efficiency was high (76.0–81.7%) for liposomes using ^64^Cu (Supplementary Data: Table S1). PEGylation of the particles had little effect on its complexing properties. Control liposomes **Q** and **R**, without inclusion of the DOTA-lipids, showed minimal (<6%) ^64^Cu uptake. Labelling efficiency with ^111^In (Supplementary Data: Table S2) was high for liposomes **L** (78.4%) and **M** (88.0%) containing **DEG3SL**, with no significant difference between the DODEG4-shielded (**L**) and non-shielded (**M**) formulations. The labelling efficiency was lower (36.6%) for liposome **O** containing **DEG6SL**.

For *in vivo* injections, liposomes **L**, **N**, **O** and **P** were prepared using probe sonication in order to obtain consistently small particles, with the size and zeta potential being measured prior to labelling with ∼45 Mbq ^111^In and purification using a PD10 column. All liposome samples had low polydispersities, with the DODEG4 liposomes **L** and **O** being smaller and with a greater surface charge than the DSPE-PEG2000 liposomes **N** and **P**.

### 3.6 In vivo SPECT imaging of In-111 labelled liposomes

**Fig. 3:**
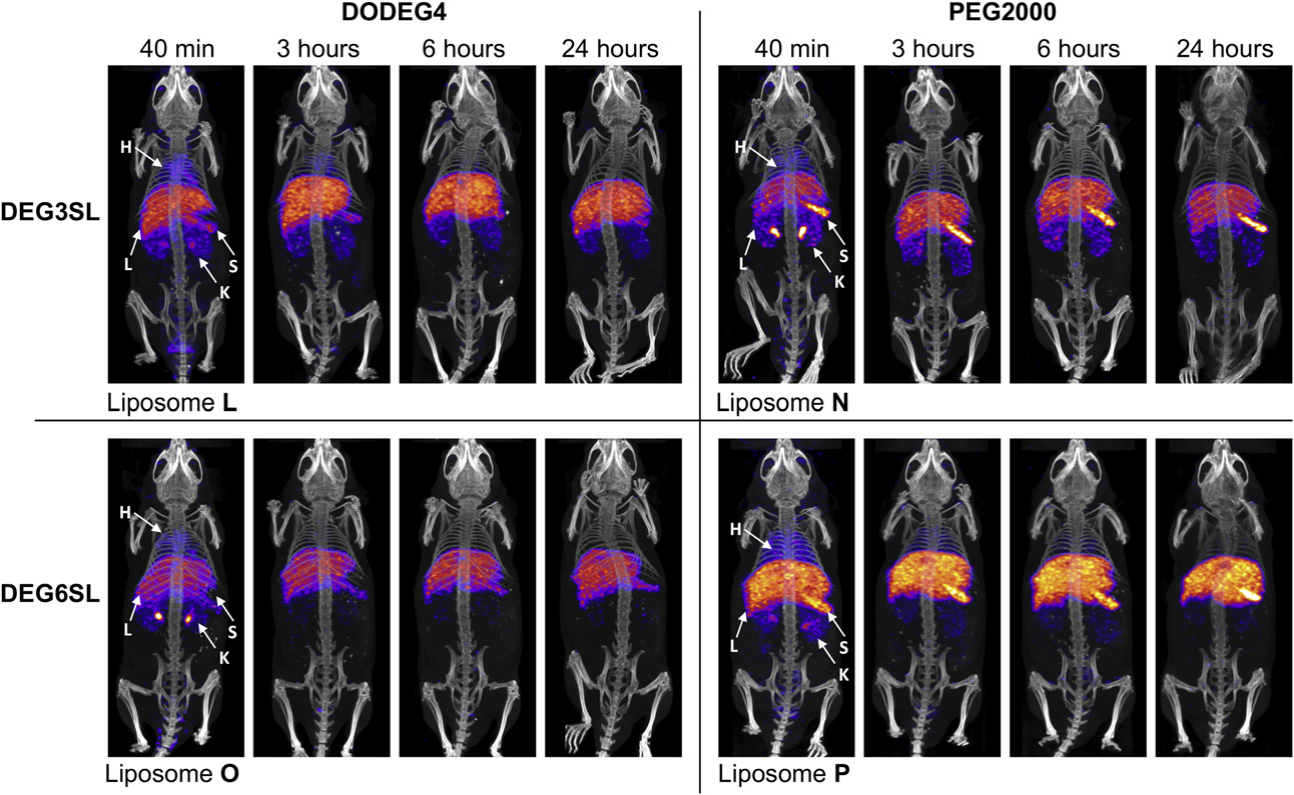
In vivo SPECT/CT distributions derived from an intravenous administration of ∼10MBq labelled DODEG4 (left hand column) liposome preparations **L** and **O**, formulated with **DEG3SL** and **DEG6SL** respectively, compared to the control DSPE-PEG2000 (right hand column) liposome preparations **N** and **P** formulated with **DEG3SL** and **DEG6SL** respectively, over the course of 24 h (H – heart, L – liver, S – spleen, and K – kidney). The bladder has been removed from the 40 min and 3 h images, allowing the images to be scaled so they were comparable to each other.

**Fig. 4:**
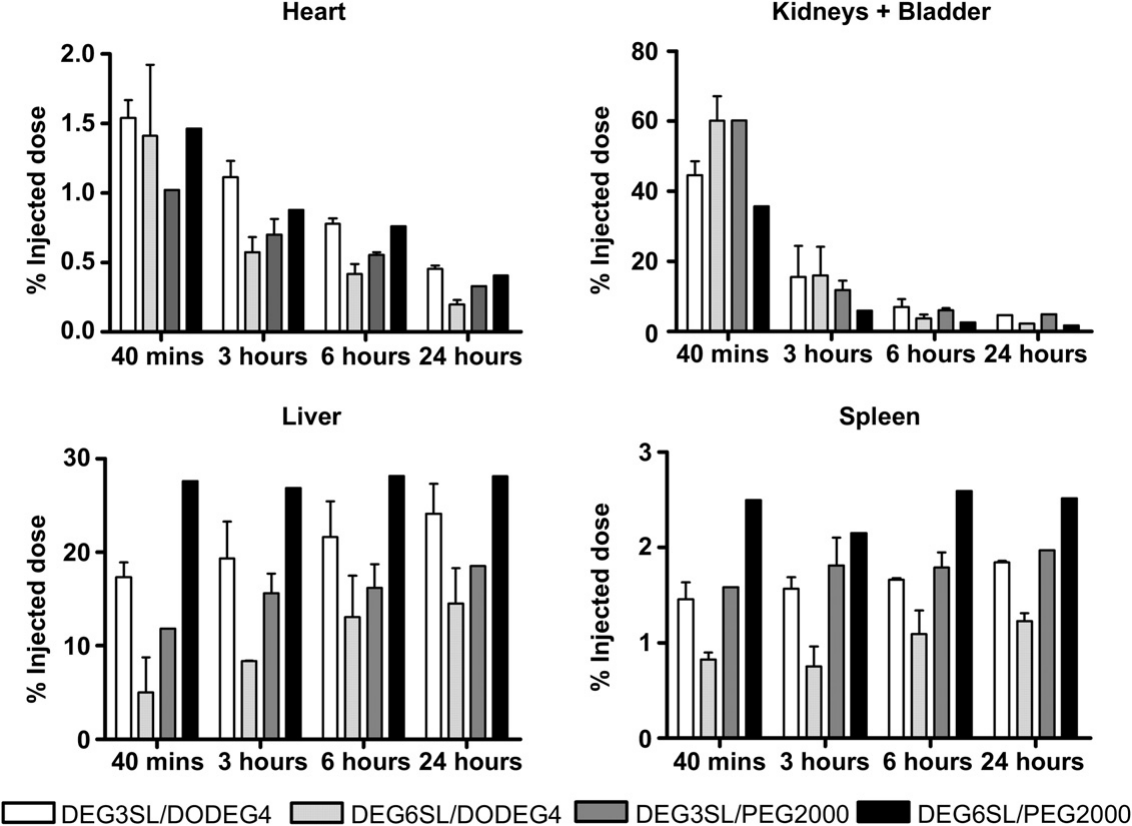
The radioactivity derived from each organ at each time point for liposomal preparations **DEG3SL/DODEG4** (**L**), **DEG6SL/DODEG4** (**O**), **DEG3SL/DSPE-PEG2000** (**N**), and **DEG6SL/DSPE-PEG2000** (**P**). This is expressed as a percentage of whole body activity at 40 min post-injection.

DODEG4 liposomes **L** (**DEG3SL**) and **O** (**DEG6SL**) as well as PEG2000 liposomes **N** (**DEG3SL**) and **P** (**DEG6SL)** were injected intravenously to assess changes in distribution patterns over a 24 h time course (Figs. 3 and 4). The bladder was removed from the images (Fig. 3) at both the 40 min and 3 h time points so that the thresholding of the uptake in the internal organs could be compared. However, the data for the kidneys and bladder show that almost half the radioactivity injected (∼40% liposomes **L** & **P** and ∼60% liposomes **N** & **O**) is being excreted at the 40 min time point (Fig. 4). At 3 h there was still some radioactivity in the bladder for all liposome formulations but not at later time points although the kidneys appear to retain some activity throughout (Figs. 3 and 4). All liposome preparations also showed rapid uptake within the liver and spleen even at the 40 min time point. Liposomes **L** and **P** had a much higher uptake at 40 min compared to **N** and **O**, which may explain the slight reduction in their renal clearance at this time point (Figs. 3 and 4). Liposome **P** also had the highest uptake within the spleen, although both liposomes **L** and **N** presented fairly similar uptakes. In the images, both of the PEG2000 liposomes appear to show a characteristically high uptake in the spleen compared to both DODEG4 liposomes. This may be indicative of specific uptake into a select cell type within the spleen for these liposomes (Figs. 3 and 4). After the initial uptake, at 40 min post-injection further uptake in both the liver and spleen for most liposome preparations appeared to be gradual over the 24 h as indicated by the gradual decrease in radioactivity taken from the heart (indicative of blood pool) which was similar in rate for all liposomes (Figs. 3 and 4). This suggests that the liposomes have similar half-lives and that it is therefore differences in uptake and excretion that mostly characterises them. As liposome **P** (**DEG6SL**/PEG2000) had the highest uptake in the liver and spleen followed by **L** (**DEG3SL**/DODEG4) with liposomes **N** (**DEG3SL**/PEG2000) and **O** (**DEG6SL**/DODEG4) having the lowest, there is no clear correlation in the biodistribution that is characteristic to either the two different PEG coatings or to the In-DOTA lipids.

## 4 Discussion

The aim of this study was to develop and optimise multifunctional liposomes for a range of imaging modalities [26], and simultaneously to act as effective passive delivery agents for disease states such as tumour delivery via the EPR effect. In previous work, liposomal formulations incorporating DSPE-PEG2000 have been prepared for *in vivo* imaging or delivery applications. The resulting large, bulky PEG coating is believed to shield the liposomes, reducing their clearance *via* the RES, resulting in a long blood half-life and significant uptake within the interstitial space of subcutaneous tumours over 24 h [2,3,5,6]. However, liposomes formulated with the high percentages of DSPE-PEG2000 (7–10%) required for *in vivo* stability are poorly taken up into cells *in vitro* [7], reducing the efficiency of delivery at the target tissue and leading to uptake in unwanted tissues.

In the present study, we sought to combat these limitations of PEG2000 shielding by formulating liposomes including a high proportion (50%) of a cationic lipid, DODEG4 [10], bearing short *n*-ethylene glycol units. We envisaged that this would lead to liposomes with a uniform coverage of shallow PEG shielding, which would still confer a long blood half-life without impairing cellular uptake. To make the liposomes multifunctional for imaging we have prepared lipids containing a chelator (DOTA) in the head group, for the chelation of paramagnetic Gd^3+^ for MRI, and the radionucleides ^111^In and ^64^Cu for SPECT and PET respectively, and have co-formulated these with DODEG4, DOTMA, DOPE and fluorescent lipids. As the short oligoethylene glycol spacers on the DODEG4 may interact with functional groups on the other lipid components of the liposomes, three DOTA lipids, **DEG1SL** (**15**), **DEG3SL** (**16**) and **DEG6SL** (**17**), were synthesized with different length oligoethylene glycol spacer units between DOTA and the rest of the lipid. For MRI imaging, the DOTA lipids were chelated with Gd^3+^ to give **Gd-DEG1SL, Gd-DEG3SL** and **Gd-DEG6SL** respectively, and liposomes were then formulated with the Gd-lpids (Table 1). For PET/SPECT imaging, liposomes were first formulated from the DOTA lipids themselves and the radiotracers subsequently chelated to the DOTA group post-formulation (Table 2). The qualities of the resulting liposomes as imaging tracers, their uptake in a range of tumour cell lines *in vitro*, and finally their *in vivo* distribution, were evaluated and compared to similar liposome incorporating DSPE-PEG2000 as a control.

Formulation with **Gd-DEG1SL, Gd-DEG3SL** and **Gd-DEG6SL** resulted in liposomes with *T*_1_ relaxivities comparable to that of the Gd.DOTA contrast agent Dotarem, commonly used in clinical imaging applications. Liposomes were also evaluated with and without the incorporation of the short PEG lipid, DODEG4, as well as with PEG2000. Although there was a trend for the relaxivity to go down slightly with PEG shielding in all liposomes except the Gd-DEG6SL formulation, the PEG shielding afforded by DODEG4 did not notably change the relaxivity of the particles. It has been previously been thought that PEG incorporation increases the relaxivities of liposomal MRI contrast agents, due to additional macromolecular bulk structure, causing reduced tumbling rates and hence increased Gd-metal water contact [16,27]. However, other groups have also showed no difference in relaxivities at 1.5 T between liposomes formulated with [49] and without [50] M-PEG2000-PE.

In this study, the most influential factor in liposome relaxivity was the length of the oligoethylene glycol spacer units between the DOTA and the lipid head group. The lipid with the shortest oligoethylene glycol spacer (**Gd-DEG1SL**) had an increased relaxivity compared to Dotarem, whereas the longest (**Gd-DEG6SL**) had the greatest reduction in relaxivity. Previous studies of liposomes formulated from other Gd-lipids, such as Gd.DOTA.DSA, have also been shown to have an enhanced relaxation compared to commercially available Gd-chelates [16,19]. This effect appears to depend on the Gd chelate being rigidly fixed to the exterior surface of the liposome, such that the nanocomplex rotates as a rigid body thus reducing the rotational tumbling rate of these large nanocomplexes [51]. Other strategies to restrict the rotational motion of the Gd-chelates in liposomal preparations, such as attaching the DOTA directly to the alkyl chains [52] have also lead to enhanced relaxivities. In our work, the DOTA chelate in Gd-DEG6SL has the most motility away from the polar head group. This local motion reduces the effective rotational tumbling rate and therefore lowers relaxivity, as has been shown for other liposomal Gd-chelates [50]. In contrast, other studies of polymerised liposomes formulated with Gd-chelates have shown that a longer linker between the Gd-chelate and the lipid head group can actually lead to higher molar relaxivities. This was attributed to greater separation between the metal ion and the surface of the liposome, allowing improved aqueous accessibility to the Gd^3+^ ion [53]. Gd^3+^-DTPA-bisamides have also been prepared possessing different alkyl chain lengths where a double bond in the alkyl chain, compared to a saturated chain, gave a more efficient MRI contrast reagent due to greater chain mobility and resulting water exchange [54]. However, in the present study the water exchange rate is not so critical for achieving a high *r*_1_ at higher field strengths [55]; the range between the three Gd-lipids is minimal and **Gd-DEG6SL** is still very much classed as a functional contrast agent, although there may be a greater difference between liposomes formulated from the three Gd-lipids at lower fields [49]. It must also be noted that the relaxivities for each formulation was measured as a function of Gd concentration to assess how our formulation affects relaxivity. However, as the liposome contains numerous DOTA chelators (30%) the Gd payload per liposome [16] is increased and thus the Gd relaxivity per liposome is actually much higher.

Chelation of the radionucleides ^111^In and ^64^Cu for SPECT and PET was carried out after formulation of the liposomes. The degree of chelation of the radionucleides therefore gives information on how the PEG coating affects the chelating efficiency of the DOTA. This was carried out for formulations **H**–**P**. **DEG3SL** and **DEG6SL** liposomes were evaluated with and without the short PEG lipid, DODEG4, for ^64^Cu, and **DEG3SL** and **DEG6SL** liposomes were evaluated with both DODEG4 and DSPE-PEG2000 for ^111^In, as the binding would be thought to be consistent for both metals. As our initial studies with Gd^3+^ appeared to indicate that a shorter oligoethylene glycol spacer would lead to reduced availability of the DOTA within the PEG layer that would hinder binding, **DEG1SL** formulations were not evaluated. Liposome labelling for both ^111^In and ^64^Cu was high (∼80%) although slightly lower for **DEG6SL** formulations than **DEG3SL**. This may again be due to the flexibility of the longer oligoethylene glycol spacer, causing folding of the head group into the bilayer and reducing accessibility for binding. These labelling efficiencies of ^111^In are slightly lower than those obtained in a recent study by de Vries *et al.* [21] although these were heated at 50 °C rather than 37 °C, which may aid in maximal chelation. The **DEG3SL**/DSPE-PEG2000 liposome **N** and **DEG6SL**/PEG2000 liposome **P** also showed similar binding efficiencies to the **DEG6SL**/DODEG4 formulation **O**, when assessed by PD10 purification prior to *in vivo* imaging studies, indicating that PEG does not inhibit radiolabeling of DOTA on the surface of liposomes. Therefore, these liposomes are suitable nuclear agents for both SPECT and PET scanning.

The *in* v*itro* uptake experiments showed that the three different cancer cell types all labelled and internalised each of the Gd chelated liposome formulations **A**, **B** and **C** containing the oligoethylene glycol spacer lipid DODEG4, although to differing degrees. Internalization was characterised by fluorescence microscopy as circular deposits within the cell, consistent with uptake within vesicles within the cell. In general, both the MDA-MB-231 and HeLa cells were fairly tolerant to liposome labeling with the MDA-MB-231 cells showing the highest uptake with the greatest reduction in T1, whereas the OVCAR-3 cells appeared to be the most susceptible to cell death but also had the lowest uptake corresponding to a low reduction in T1. The **Gd-DEG3SL** liposome **B** was the best tolerated but also showed the lowest uptake. Liposomes **A** and **C** were less tolerated but showed a much higher uptake in cells by both fluorescence microscopy and FACS results. Therefore the degree of uptake is affecting cell viability especially at the higher concentrations in these formulations. We have confirmed this by the dose dependent effect that can be seen by using serial dilutions of **Gd-DEG3SL** and cell viability. The equivalent non-shielded liposomes **E**, **F** and **G**, formulated with DOTMA rather than DODEG4, showed similar results by FACS to the results for **A**, **B** and **C**. Cellular uptake was also not hindered by serum within the incubation media; indeed, the presence of serum actually enhanced the cellular uptake, increasing fluorescence and also reducing the rate of uptake, resulting in similar cell viabilities as the shielded liposomes at 2 h rather than 4 h incubation. This may result from the serum causing the particles to stick to the cell surface, or in the presence of serum uptake of aggregates is also seen. However, the DSPE-PEG2000 liposome, **D**, showed no cellular internalization, and although the FACS results showed that the percentage of cells that were labelled by liposome **D** was similar to the three DODEG4 liposome formulations **A**, **B** and **C**, the degree of shift in fluorescence was much lower. In fact, when cells labelled with liposome **D** were visualised by fluorescence microscopy, the fluorescence appeared as a dull halo around the cells, indicating that the liposomes were only attached to the outside of the cell and had not been internalised.

Formulations of bimodal cationic liposomes containing Gd-lipids and fluorescence markers, but without PEG shielding, have already been shown to have good uptake properties within HeLa cells with little effect on cell viability [16]. Such liposomes have also proven to be effective for siRNA delivery *in vivo* to OVCAR-3 cells [17]. However, these papers did not compare the uptake of these non-shielded liposomes with liposomes containing PEG2000 in their *in vitro* experiments; rather, different formulations were utilised for the *in vivo* experiments, making a comparison of the effectiveness of liposomes coated with PEG2000 with non-shielded liposomes impossible [16,17]. We hypothesise that the limited uptake of the PEG2000-containing liposome **D** is most likely due to two factors. Firstly, the long neutral PEG2000 lipid hides the charge of the liposome, which affects cell uptake, as cells have been shown to preferentially take up charged particles. Secondly, PEG2000 is hydrophilic in character, which decreases the adsorption of serum proteins and opsonins to the surface of the liposome bilayer, resulting in limited interaction with cell walls, also reducing cellular uptake [1]. For this reason, stealth liposomes formulated with PEG2000 lipids generally require the use of targeting ligands for enhanced cellular uptake [6,7]. Our results show that the use of the lipid DODEG4, with a short oligoethylene glycol spacer, for the preparation of shielded liposomes, appears to limit this inhibiting effect on cellular uptake when compared to liposomes formulated incorporating DSPE-PEG2000. The differences in uptake for the different Gd-lipid formulations **A**, **B** and **C** may relate to how prominent the neutrally charged Gd-DOTA complex is on the surface. The relaxivity measurements may suggest that the Gd-DOTA in the **DEG1SL** liposome, **A**, is buried within the DODEG4 shielding layer, as the oligoethylene glycol spacers are about the same length. Similarly, the ^111^In chelating experiments may indicate that the DOTA chelates in the **DEG6SL** liposome **C** are folded back into the oligoethylene glycol layer. However, the observed differences in uptake are not correlated to the surface charge of these particles, with liposomes **B** and **C** having similar positive zeta potentials and liposome **A** having the most positive surface charge, nor are they related to the size of the particles, with liposomes **C** and **D** having significantly greater hydrodynamic sizes than liposomes **A** and **B**.

The pharmacokinetic properties of liposomes **L**, **O**, **N** and **P,** as assessed by SPECT/CT, were also markedly different. Directly after injection and at 3 h all liposome preparations showed uptake in the bladder although free indium had been removed by PD10 purification. This may be partly due to the distribution of size within the liposome formulations, with smaller sizes excreted via the kidney. At 40 min post-injection uptake in the liver and spleen could be seen for all liposome preparations, which then gradually increased over the 24 h time course. The ^111^**In-DEG6SL**/DSPE-PEG2000 (**P**) showed the highest liver and spleen uptake followed by ^111^**In-DEG3SL**/DODEG4 (**L**), whereas both the ^111^**In-DEG3SL**/DSPE-PEG2000 liposome (**N**) and ^111^**In-DEG6SL**/DODEG4 liposome (**O**) were shown to have a reduced uptake in the liver. However, liposome **N** did exhibit a high uptake in the spleen, with a characteristic uptake pattern in images that was consistent for both DSPE-PEG2000 liposomes. The gradual increase in uptake over the 24 h indicates that a specific fraction remains in the blood pool at the earlier time points. The reduction in blood concentration is shown by the heart values over the 24 h and indicates that all of the liposomes have fairly similar clearance rates. The uptake and clearance differences between liposomes unfortunately cannot currently be attributed to a specific component of the liposomal formulation. There is no correlation between the degree of uptake in liver and spleen that relates to either the changes in PEG shielding between the DODEG4 and DSPE-PEG2000 formulations, or to the differences in *n*-EG linker lengths between the In-DOTA lipids in this study, although the images for liposomes formulated with DSPE-PEG2000 do appear to show characteristic uptake in the spleen.

These results are highly significant, as they indicate for the first time that it may be possible to “tune” liposomes to change their distribution *in vivo*, as we have shown fairly substantial changes by just adjusting the depth and coverage of the PEG shielding and matching this to the length of PEG spacers between the component lipids and the functional groups attached to them. They also indicate that, contrary to other literature in this area, shielding with PEG2000 lipids is not always the predominant factor governing distribution of liposomes *in vivo*, as liposomes **N** and **P** show very different behaviours. Although the distributions differed between formulation the blood clearance, as judged by activity in the heart, was fairly similar. Therefore it is hard to predict differences which might occur in the degree of uptake of these different liposomes in tumour models by EPR. However, we have also shown that liposomes formulated with DODEG4 shielding have a high cell uptake *in vitro*, whereas liposomes formulated with DSPE-PEG2000 do not. This would mean that drug delivery for these formulations would be more efficient and therefore deliver an effectively higher payload to target cells, compared to DSPE-PEG2000 formulations, when delivered passively. Further work will be required to determine the relationships between the depth and coverage of the *n*-EG or PEG shielding, the length of *n*-EG spacer between lipid and functional group, and the biodistribution and EPR uptake of these formulations.

Remarkably little previous work has been carried out to investigate the overall biodistribution of bimodal liposomes for imaging, or the effects of changes in formulation on biodistribution. Although the incorporation of radionuclides should allow for these effects to be studied, to date no SPECT investigations of liposome biodistribution have been published, with the exception of a key study by Boerman *et al* [23] looking at the uptake of encapsulated ^111^In PEGylated liposomes, which interestingly also showed a high uptake of liposomes within the spleen. Similarly, although the inclusion of a paramagnetic label should make MRI studies feasible, only liver uptake has so far been quantified by analysing tissue T_1_ at longitudinal time points for Gd-liposome formulations [16]. The majority of reported biodistribution data has been acquired post mortem by either fluorescence [16–18], or Gd distribution [56], or Geiger counting [23] measurements.

## 5 Conclusions

We have prepared a series of chelating lipids with oligoethylene glycol spacers of differing lengths between the DOTA chelator and the lipid head group, and used these to formulate multifunctional liposomes, bearing paramagnetic or radiotracer ions, and also bearing fluorophore lipids. We have shown that these multimodal liposomes can be developed as functional MRI contrast agents similar to those previously shown in the literature, as well as radionuclide tracers for both PET and SPECT. When co-formulated with DODEG4, a lipid with a short *n*-EG spacer, to give shielded liposomes with a shallow but even coverage of PEG, these non-targeted liposomes showed good cellular internalization in a range of tumour cells compared to the limited cellular uptake of liposomes formulated with DSPE-PEG2000. Moreover, by matching the depth of PEG coverage afforded by DODEG4 to the length of the *n*-EG spacers of the DOTA lipids, we have shown that similar distributions and blood half-lives to DSPE-PEG2000-stabilised liposomes can be achieved. We envisage that liposomes functionalized in this manner should eventually be capable of delivering high payloads of therapeutic drugs to tumour cells, not just *in vitro* but also *in vivo*, without affecting the biodistribution characteristics shown by liposomes modified by long chain PEG, but with an enhanced capability for cellular uptake and internalization, bringing this technology one step closer to the clinic.

## Figures and Tables

**Table 1:** Compositions, hydrodynamic sizes and zeta potentials of Gd-liposomes with oligoethylene glycol shielding (**A**, **B**, **C**), stealth lipid shielding (**D**) and without PEG shielding (**E**, **F**, **G**).

Liposome	DOPE	Gd-lipid	PEG-lipid	DOTMA	FL-DHPE	DLS (PDI)	Zeta
**A**	9%	30% **Gd-DEG1SL**	50% **DODEG4**	10%	1%	96.8 nm (0.2)	(+) 62 mV
**B**	9%	30% **Gd-DEG3SL**	50% **DODEG4**	10%	1%	92.9 nm (0.2)	(+) 56 mV
**C**	9%	30% **Gd-DEG6SL**	50% **DODEG4**	10%	1%	177 nm (0.2)	(+) 56 mV
**D**	2%	30% **Gd-DEG3SL**	7% **DSPE-PEG2000**	60%	1%	146 nm (0.3)	(+) 45 mV
**E**	9%	30% **Gd-DEG1SL**	–	60%	1%	212.9 nm (0.3)	(+) 55 mV
**F**	9%	30% **Gd-DEG3SL**	–	60%	1%	220.8 nm (0.4)	(+) 53 mV
**G**	9%	30% **Gd-DEG6SL**	–	60%	1%	244.4 nm (0.3)	(+) 44 mV

**Table 2:** Compositions of liposomes labeled with ^64^Cu (**H**–**K**) and ^111^In (**L–P**). Liposomes **H**, **I, L**, **M** and **N** were formulated using **DEG3SL 16** then labelled with ^64^Cu or ^111^In post-formulation. Liposomes **J**, **K, O** and **P** were formulated using **DEG6SL 17** then labelled with ^64^Cu or ^111^In post-formulation. Liposomes **Q** and **R** were formulated as controls lacking the DOTA-lipids. Samples **L**, **N**, **O** and **P** were used for *in vivo* biodistribution.

Liposome	DOPE	Cu/In-lipid	PEG-lipid	DOTMA	FL-DHPE	DLS (PDI)	Zeta
**H**	10%	30% **Cu-DEG3SL**	50% **DODEG4**	10%	–	–	–
**I**	10%	30% **Cu-DEG3SL**	–	60%	–	–	–
**J**	10%	30% **Cu-DEG6SL**	50% **DODEG4**	10%	–	–	–
**K**	10%	30% **Cu-DEG6SL**	–	60%	–	–	–
**L**	9%	30% **In-DEG3SL**	50% **DODEG4**	10%	1%	73.9 nm (0.2)	(+) 54 mV
**M**	10%	30% **In-DEG3SL**	–	60%	–	–	–
**N**	2%	30% **In-DEG3SL**	7% **DSPE-PEG2000**	60%	1%	141.7 nm (0.2)	(+) 43 mV
**O**	9%	30% **In-DEG6SL**	50% **DODEG4**	10%	1%	70.1 nm (0.2)	(+) 50 mV
**P**	2%	30% **In-DEG6SL**	7% **DSPE-PEG2000**	60%	1%	142.6 nm (0.2)	(+) 31 mV
**Control Q**	40%	–	50% **DODEG4**	10%	–	–	–
**Control R**	50%	–	–	50%	–	–	–

**Table 3:** Comparison of relaxivities (*r*_1_) for PEGylated and non-PEGylated liposomes.

Liposome	Gd-lipid	PEG shielded	*r*_1_/mm^−1^ s^−1^
**A**	**Gd-DEG1SL**	DODEG4	2.95
**B**	**Gd-DEG3SL**	DODEG4	2.29
**C**	**Gd-DEG6SL**	DODEG4	1.97
**D**	**Gd-DEG3SL**	DSPE-PEG2000	2.12
**E**	**Gd-DEG1SL**	–	3.05
**F**	**Gd-DEG3SL**	–	2.37
**G**	**Gd-DEG6SL**	–	1.78
