# Supplementary material for: Incorporation of paramagnetic, fluorescent and PET/SPECT contrast agents into liposomes for multimodal imaging
Source: Biomaterials. 2013 Jan;34(4):1179–92. doi: 10.1016/j.biomaterials.2012.09.070 (PMC3520009; doi:10.1016/j.biomaterials.2012.09.070)
Supplement: Supplementary file 1 [file mmc1.doc]

**Incorporation of Paramagnetic, Fluorescent and PET/SPECT Contrast Agents into Liposomes for Multimodal Imaging**

**Supporting Information**

*Nick Mitchell, Tammy L. Kalber, Maggie Cooper, Kavitha Sunassee, Samantha L. Chalker, Karen P Shaw, Katherine L. Ordidge, Adam Badar, Samuel M. Janes, Philip J. Blower, Mark F. Lythgoe, Helen C. Hailes and Alethea B. Tabor*

**Contents**

| **Figure S1** MALDI of **Gd-DEG6SL** | S2 |
| --- | --- |
| **Figure S2** MALDI of **Gd-DEG3SL** | S2 |
| **Figure S3** MALDI of **Gd-DEG1SL** | S2 |
| **Table S1** Percentage incorporation of ^64^Cu in DOTA lipids and liposomes | S3 |
| **Table S2** Percentage incorporation of ^111^In in DOTA lipids and liposomes | S3 |
| **Figure S4** FACS analysis of cellular liposome uptake | S4 |
| **Table S3** FACS percentages for liposome incubations **A** - **G** | S5 |
| **Figure S5** Confocal microscopy of cellular liposome uptake of liposomes **E** - **G** | S5 |
| **Figure S6** Cellular liposome uptake of liposomes **B** and **C** in the presence and absence of serum | S6 |
| **Figure S7** Cell viability data from Trypan Blue counts | S7 |

**Figure S1 MALDI-MS of** **Gd-DEG6SL - 1495 g.mol^-1^**

**Figure S2 MALDI-MS of** **Gd-DEG3SL - 1363 g.mol^-1^**

**Figure S3 MALDI-MS of** **Gd-DEG1SL - 1274 g.mol^-1^**

| **Sample** | **^64^Cu (% bound)** |
| --- | --- |
| **DEG3SL** | 96.4 |
| **DEG6SL** | 77.3 |
| Liposome **H** | 77.5 |
| Liposome **I** | 76.0 |
| Liposome **J** | 78.5 |
| Liposome **K** | 81.7 |
| Control liposome **Q** | 5.8 |
| Control liposome **R** | 0.0 |

**Table S1 Percentage incorporation of ^64^Cu in DOTA-lipids and liposomes, measured by ITLC.**

| **Sample** | **^111^In (% bound)** |
| --- | --- |
| **DEG6SL** | 72.0 |
| Liposome **L** | 78.4 |
| Liposome **M** | 88.0 |
| Liposome **O** | 36.6 |

**Table S2** Percentage incorporation of ^111^In in DOTA-lipids and liposomes, measured by ITLC.


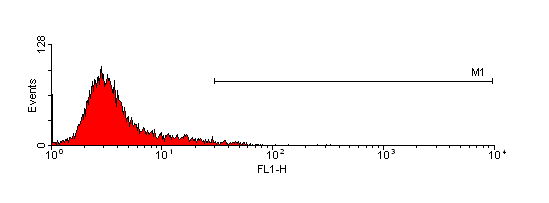

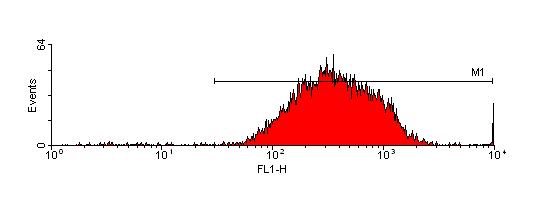

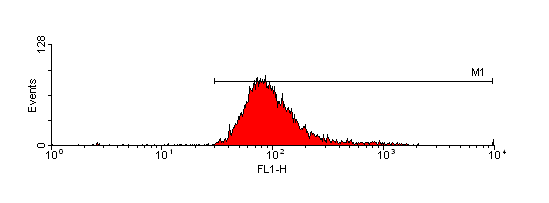


98.81 %

97.95 %

3.24 %

Control

DODEG4

PEG2000

**Figure S4:** FACS histogram plots for control HeLa cells, HeLa cells treated with Liposome **B** (containing DEG3SL and the PEG lipid DODEG4), and HeLa cells treated with Liposome **D** containing the DEG3SL and DSPE-PEG2000 lipid. Note the shift in fluorescence to the left for the DSPE-PEG2000 liposome.

| Liposome | Gd-Lipid | PEG-Lipid | HeLa | OVCAR-3 | MDA-MB-321 | MDA-MB-231 Serum + |
| --- | --- | --- | --- | --- | --- | --- |
| Control cells | - | - | 3.24 ± 0.18 | 2.56 ± 0.08 | 2.38 ± 0.04 | 2.28 ± 0.05 |
| A | DEG1SL | DODEG4 | 96.64 ± 0.03 | 94.30 ± 0.04 | 96.40 ± 0.07 | - |
| B | DEG3SL | DODEG4 | 98.81 ± 0.02 | 98.72 ± 0.02 | 99.40 ± 0.15 | 96.44 ± 1.04 |
| C | DEG6SL | DODEG4 | 96.88 ± 0.03 | 99.27 ± 0.07 | 98.08 ± 0.41 | 97. 34 ± 0.25 |
| D | DEG3SL | PEG2000 | 97.95 ± 0.01 | 98.81 ± 0.00 | 95.85 ± 0.11 | - |
| E | DEG1SL | - | - | - | 98.74 ± 0.33 | - |
| F | DEG3SL | - | - | - | 99.04 ± 1.04 | - |
| G | DEG6SL | - | - | - | 91. 63 ± 1.65 | - |

**Table S3**: FACS percentages for liposome incubations **A-G**. ± = standard deviation.


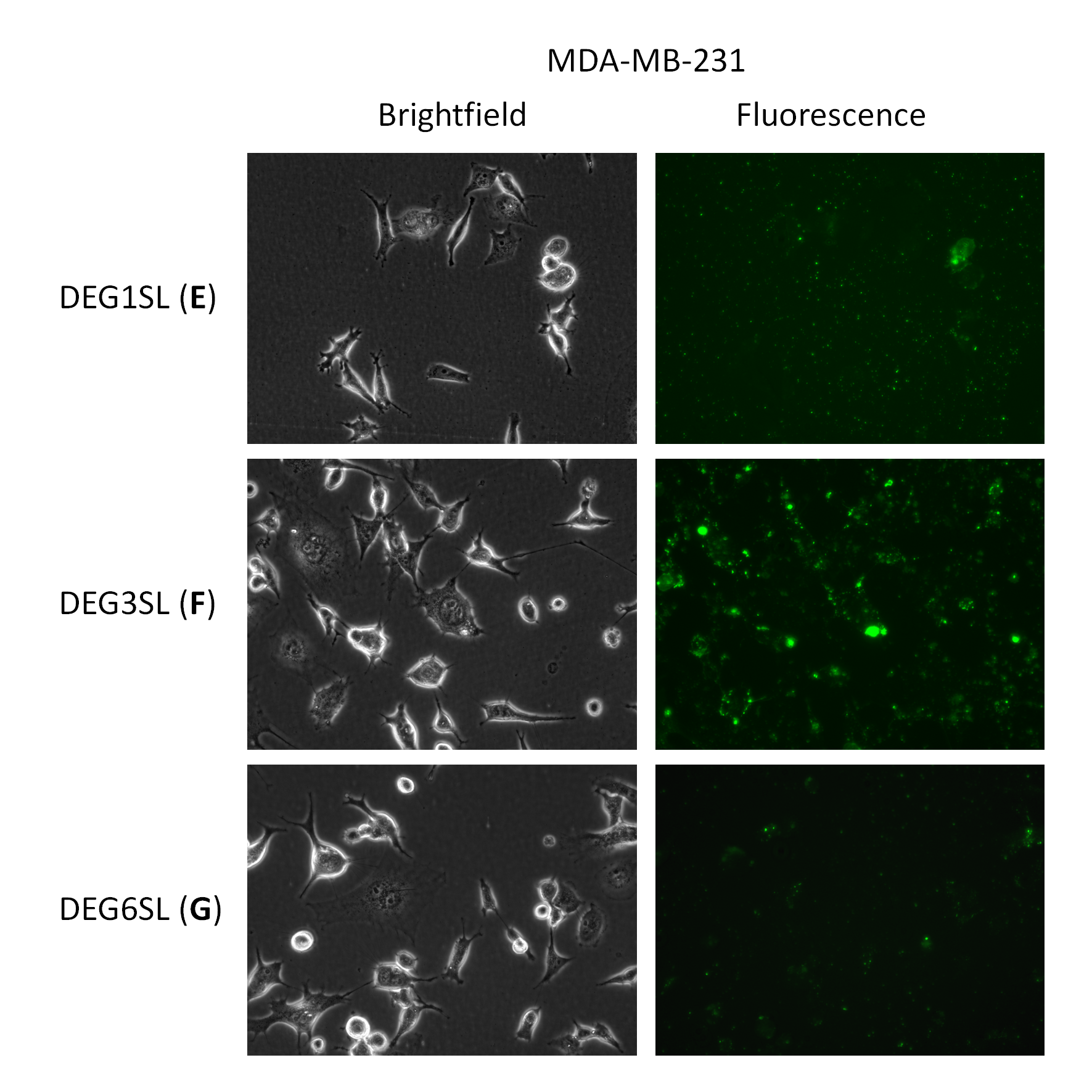


**Figure S5:** Brightfield and corresponding fluorescent image for liposomes **E-G** containing DEG1SL, DEG3SL, or DEG6SL but with no DODEG4 (x100 magnification).


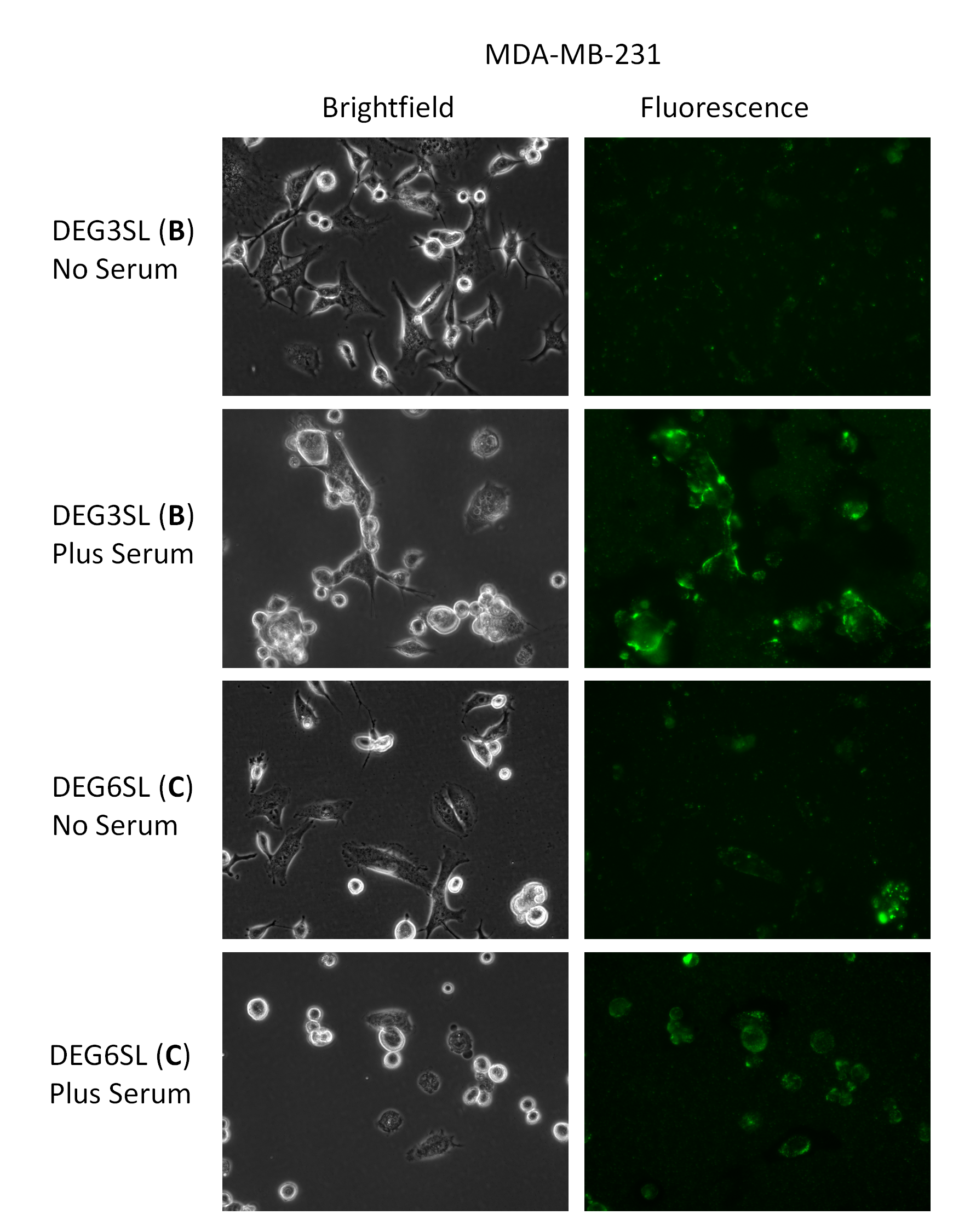


**Figure S6:** Brightfield and corresponding fluorescence image for liposomes **B** and **C** containing DEG3SL and DEG6SL respectively, both containing DODEG4 and incubated in either serum free or normal culture medium (x100 magnification).

**Figure S7:** Cell viability data from Trypan Blue counts for cells labelled with serial dilutions of liposome B containing DEG3SL and DODEG4 lipid, and Liposome formulations **A**, **B**, **C** and **D** containing the DEG1SL, DEG 3SL, DEG6SL (all with DODEG4) and DEG3SL with DSPE-PEG2000 respectively for all tumour cell lines.
